# Constitutive overexpression of *Qui-Quine Starch* gene simultaneously improves starch and protein content in bioengineered cassava (*Manihot esculenta* Crantz)

**DOI:** 10.3389/fpls.2024.1442324

**Published:** 2025-02-24

**Authors:** Bertrand Bachaumond Hankoua, Marieme Diao, Ayalew Ligaba-Osena, Rafael A. Garcia, Sarahani Harun, Yogesh K. Ahlawat

**Affiliations:** ^1^ Food Biotechnology Laboratory, Department of Human Ecology, College of Agriculture, Sciences and Technology, Delaware State University, Dover, DE, United States; ^2^ Research & Development Division, Reckitt Benckiser Inc, Evansville, IN, United States; ^3^ Laboratory of Plant Molecular Biology and Biotechnology, Department of Biology, The University of North Carolina at Greensboro, Greensboro, NC, United States; ^4^ Dairy and Functional Foods Research Unit, Agricultural Research Service, United States Department of Agriculture, Wyndmoor, PA, United States; ^5^ Centre for Bioinformatics Research, Institute of Systems Biology (INBIOSIS), Universiti Kebangsaan Malaysia (UKM), Bangi, Selangor, Malaysia; ^6^ University Centre for Research and Development, Chandigarh University, Mohali, India; ^7^ Centre of Research Impact and Outcome, Chitkara University Institute of Engineering and Technology, Chitkara University, Rajpura, India

**Keywords:** cassava, protein biofortification, protein deficiency, overexpression, QQS, starch improvement, food security, malnutrition

## Abstract

Cassava is a crucial source of daily calorie intake for millions of people in sub-Saharan Africa (SSA) but has an inferior protein content. Despite numerous attempts utilizing both traditional and biotechnological methods, efforts to address protein deficiency in cassava have yet to meet with much success. We aim to leverage modern biotechnologies to enhance cassava's nutritional value by creating bioengineered cassava cultivars with increased protein and starch content. In this study, we utilized *Qui-Quine Starch* (*QQS*), a novel orphan gene unique to *Arabidopsis thaliana*, to develop transgenic cassava plants with increased protein and starch accumulation in their tissues. A total of 10 independent transgenic cassava lines expressing *QQS* were successfully regenerated in this study, among which line R7 (F) demonstrated superior growth vigor. Quantitative RT-PCR verified the expression of the *QQS* gene in the transgenic lines. Data showed that *QQS* expression in cassava plants increased leaf protein content by 36% in line R’’’ (LA) L2 and root protein by 17% for the same line compared to their wild-type and empty vector (NPTII) control plants. Moreover, leaf-soluble total carbohydrates increased by 51.76% in line R (G) L2, and root-soluble total carbohydrates increased by 46.75% in line R7 (F). The novel function of *QQS* in increasing the starch content in the transgenic biomass is demonstrated. No significant change in the content of specific amino acids was observed among the lines and various plant parts. In addition, *QQS* expression revealed increased biomass, plant vigor, and early In vitro mini-tubers production for line R7 (F). Gene interaction study between AtQQS and 59 interacting partners generated 184 interactions or edges. These gene networks comprised several functional categories regulating the starch metabolic and auxin biosynthetic processes. The role of *QQS* in imparting starch and protein content of transgenic cassava plants is validated. The next logical step is the evaluation of biochemical profiles of cassava lines expressing *QQS* that reach maturity and the transferability of these findings to consumer-preferred cassava cultivars and local landraces grown in SSA. This study represents the first biotechnological report demonstrating a simultaneous increase of protein and starch content in bioengineered cassava.

## Introduction

More than 270 million people suffer from chronic malnutrition and 400 million from micronutrient deficiencies in Sub-Sahara Africa (SSA), where subsistence farmers and their families are most at risk from the implications of undernutrition (Graham and Welch, 1996; Harika et al., 2017; Fraval et al., 2019). Many of these farmers, 250 million and more of the poorest people in the world, rely on cassava as their staple food (Narayanan et al., 2019). Cassava roots’ starch provides over 25% of dietary energy for more than 250 million Africans (Sayre et al., 2011). Children consuming cassava as a staple food are at risk for inadequate zinc, iron, and vitamin A intake (Gegios et al., 2010; Narayanan et al., 2019). Moreover, a typical cassava-based diet provides less than 30% of the minimum daily requirement for protein and only 10%–20% for iron, zinc, and vitamin A (Sayre et al., 2011). Although cassava is regarded as a food security crop in many developing countries, it has crucial deficiencies in essential minerals, vitamins, and protein. If one’s diet primarily relies on staple foods like cassava, there may be adverse health consequences unless the diet is enriched with essential nutrients. Many individuals who rely solely on cassava for their dietary energy are poor and cannot afford supplementary foods to meet their protein needs. Consequently, enhancing the nutritional profile of cassava crops has emerged as a pressing concern for scientists, particularly cassava breeders, and biotechnologists, to ensure that those dependent on it obtain their daily nutrient intake.

To effectively address the issue of agricultural development in developing countries, it is critical to recognize the consumer-preferred crops and, most importantly, the staple crops that provide most of the calories. In (SSA), the starchy root crop cassava ranks first in total productivity and number two behind maize as a source of calories (FAO/WHO, 2002; FAO, 2012; FAO, 2013; Narayanan et al., 2019). For cassava, starch content represents 64-72 percent of its total carbohydrates, depending on the cultivars or genotypes. Classical and advanced molecular breeding approaches of cassava for increasing the overall biomass and enhanced starch content of root tuber have represented, over the years, the fundamental objective of various cassava improvement programs. Through these breeding objectives, cassava breeders in various cassava-producing countries worldwide have developed and adopted novel cassava cultivars with increased starch yield, quality, and overall biomass (Hillocks et al., 2002; Jennings and Iglesias, 2002; Malik et al., 2020; Amelework and Bairu, 2022). The application of biotechnological approaches for improving starch content and overall biomass in cassava has been attempted with some success. Starch production was increased by enhancing the activity of ADP-glucose pyrophosphorylase (AGPase) activity in transgenic cassava tuberous roots (Ihemere et al., 2006). To achieve this result, a modified form of the bacterial glgC gene by site-directed mutagenesis (G336D) was expressed under the control of a Class I patatin promoter in transgenic cassava plants (Ihemere et al., 2006). Data demonstrated that modified cassava plants exhibiting the highest level of AGPase activities had a 2.6-fold increase in total tuberous root biomass under greenhouse growing conditions. Additionally, these modified cassava plants with the highest AGPase activity in their tuberous root showed significant increases in their above-ground biomass (Ihemere et al., 2006). Additionally, cassava is known for its high level of cyanogen, which may render the crop poisonous for daily consumption (Chavarriaga-Aguirre et al., 2016). Breeding programs have attempted to overcome the high levels of cyanogen, viral diseases, and nutrient deficiencies in cassava by trying conventional and transgenic breeding methods (Chavarriaga-Aguirre et al., 2016). The amino acid content of cassava reveals a shallow composition of sulfur amino acids, which are essential for human health (Omole, 1977; Nassar and Sousa, 2007). Traditional breeding methods have met little success in increasing the protein content of cassava, mainly due to the absence of cultivars with high protein content in the available local landrace germplasms for use as parents in classical and advanced breeding approaches targeting protein enhancement. This setback makes transgenic technology more attractive in achieving protein enhancement in cassava. Biotechnology has been proven successful in developing low cyanogen content (Siritunga and Sayre, 2003, 2004), insect-resistant (Ladino et al., 2002), virus-resistant (Lin et al., 2019; Narayanan et al., 2021), and herbicide-resistant (Sarria et al., 2000) cassava cultivars, and many nutritional traits such as vitamin A (Welsch et al., 2010; Failla et al., 2012), iron (Ihemere et al., 2012; Narayanan et al., 2015); and vitamin B (Li K. T. et al., 2015) have been enhanced. However, the use of biotechnological tools to increase protein content in cassava has not been conducted intensively, and a few attempts over the years have met with little success. A global initiative addresses iron, zinc, and selenium deficiencies by identifying cassava genotypes naturally rich in these nutrients. This study evaluates 20 South American cassava genotypes for their potential in biofortification, finding promising candidates like MS018, DG014, and DG839, which exhibit elevated levels of Fe, Zn, and Se alongside superior photosynthetic capacity and increased yield potential. The research contributes to developing cassava genotypes with enhanced agronomic biofortification and improved yield prospects (Inacio et al., 2024).

The first attempt was to create a strong protein sink in roots, which was shown to be sufficient to elevate total root protein. A class I patatin promoter-driven cassava hydroxy nitrile lyase (HNL) construct was used to produce transgenic plants with a three-fold increase in total root protein and an 80% reduction in root linamarin levels (Narayanan et al., 2011). Cassava hydroxynitrile lyase (HNL) is an enzyme that catalyzes the conversion of acetone cyanohydrin to cyanide. Because this enzyme is localized in the apoplastic space around leaf cells, it could presumably be induced to accumulate, thereby overexpression without turning over. The second attempt consisted of accumulating proteins in roots expressing artificial storage proteins *ASP1*, designed to be rich in essential amino acids (Zhang et al., 2003).

These previous attempts suggest that increasing protein content in staple crops such as cassava, a diet of the malnourished, is still an extremely challenging task (Narayanan et al., 2011). No cassava cultivar with high protein content has been developed to meet the minimum daily requirements (MDR). We developed a novel bioengineered cassava cultivar and identified the best transgenic plants expressing a high protein-enhancing trait. Previous studies have demonstrated that the ectopic expression of *QQ*S (Li L. et al., 2015) increased soybean protein independently of the genetic background and the original protein content of the cultivar. Furthermore, *QQS* is known to regulate the metabolic processes that affect the partitioning of carbon and nitrogen among proteins and carbohydrates, thereby resulting in a variation in leaf and seed composition (Li L. et al., 2015). Molecular and biochemical tools were employed to validate the transgenic nature of the cassava lines expressing *QQS*, and their nutritional profile was examined for protein and soluble total carbohydrate enhancement. QQS gene was expressed in the model cassava cultivar (cv 60444), which is not a preferred cultivar for farmers in Africa (Chavarriaga-Aguirre et al., 2016) however it was chosen because it is among the African germplasm for which genetic transformability has been demonstrated, and the time from *Agrobacterium* co-culture to the regeneration of whole plants is 4.5–5 months with escape rates of 5% or less (Chavarriaga-Aguirre et al., 2016). The subsequent integration of protein and starch nutritional traits into the genetic backgrounds of cassava favored by farmers in SSA (Hankoua et al., 2005, 2007; Nyaboga et al., 2013) should be attempted. This proof-of-concept demonstrated in this study using cv 60444 will guarantee critical success for delivering starch and, more importantly, protein-biofortified cassava products to millions of poor smallholder cassava farmers and consumers.

The study aimed to investigate the effects of introducing the Qui-Quine starch gene into cassava plants through genetic modification. Specifically, the study aims to assess how the constitutive overexpression of this gene influences the levels of both starch and protein content in transgenic cassava plants. Furthermore, the studies seek to elucidate the potential of this genetic modification approach to enhance cassava’s nutritional quality and yield, thereby contributing to efforts to improve food security and address malnutrition in regions where cassava is a staple crop. The orphan gene At*QQS* (*Qua-Quine Starch*) and its interacting partner, a transcription factor NF-YC4 (Nuclear Factor Y, subunit C4), have been proven to elevate the levels of protein in leaves and seeds without influencing the growth and yield of agronomically significant species (Li L. et al., 2015). Other studies, unrelated to nutritional traits enhancement, demonstrated that the increased expression of At*QQS* and NF-YC4 in Arabidopsis and soybean results in novel functionalities such as heightened resistance or decreased susceptibility to various pathogens, including viruses, bacteria, fungi, aphids, and soybean cyst nematodes (Qi et al., 2019). Furthermore, investigating the gene network involving At*QQS* and its interactor, NF-YC4 proves crucial in unravelling the intricate mechanisms underlying the interaction between At*QQS* and starch metabolic processes and its influence on auxin metabolic processes. This insight contributes to a more comprehensive understanding of the genetic regulatory pathways involved in these essential physiological processes.

## Materials and methods

### Molecular cloning and plant transformation

#### *QQS* gene synthesis and characterization of the donor plasmid

The availability of the complete nucleotide sequence of the cassava genome (Prochnik et al., 2012) prompts us to optimize the sequence of the *QQS* gene for better expression in cassava by taking advantage of the preferred codon usage of the gene expression machinery of cassava (Dai et al., 2000). This codon optimization of *QQS* could positively impact the expression of *QQS* in transgenic cassava plants. The *QQS* gene sequence (GenBank# NM_113975) was sent for synthesis and *QQS* codon optimization at Integrated DNA Technologies, Inc. (Coralville, Iowa, USA). The synthesized and codon-optimized sequence was cloned at the Sac1 and Kpn1 restriction sites in the shuttle vector pCU57, a 2.7 Kb Genscript vector having a bacteria ampicillin-resistant marker (**Supplementary Figure S1A**). The sequence alignment of the original and optimized *QQS* sequences is shown (**Supplementary Figure S2**). In **Supplementary Figure S2**, nucleotides in “red” in the optimized sequence are codons preferred by the cassava expression machinery. The sequences of the restriction sites for SacI and KpnI restriction enzymes were added at both 3’ and 5’ of primers designed to amplify the *QQS* optimized sequence (GenBank# NM_113975) from the donor plasmid pCU57. These primer modifications facilitate restriction cloning of the synthesized *QQS* gene in a pSAT1shuttle vector (Tzfira et al., 2005), as shown in (**Supplementary Figure S1B**). PCR amplification using *QQS*’s specific primers and double restriction digestion of the pCU57-*QQS* vector with restriction enzymes SacI and Kpn I New England Biolabs (MA, USA) restriction enzymes were performed to confirm the presence of the optimized *QQS* insert before cloning into shuttle vector pSAT1 and binary vector pPZP-RC2-*npt-II* for plant expression as shown in (**Supplementary Figure S1C** **).**

#### Insertion of *QQS* optimized gene into pSAT1 shuttle vector through Gibson assembly

The purpose of cloning *QQS* optimized sequence into pSAT1 shuttle vector was to assign our gene of interest with genetic elements available in pSAT1 modular vectors such as a promoter (MAS and enhanced 35S in most cases), a multiple cloning site, a homing endonuclease or rare restriction enzyme site, a translation enhancer (5’-UTR from tobacco etch virus), and a terminator sequence as shown in the (**Supplementary Figure S1B**). Gibson’s assembly method was used for this cloning after multiple unsuccessful attempts using the ligation technique. Gibson assembly method is a ligation-free cloning technique developed to increase the efficiency and accuracy of DNA assembly to create expression plasmid (Gibson et al., 2009). The optimized *QQS* sequence was redesigned using Snap gene software to assign it with 5’ and 3’ overlap sequences (30 nucleotides were added at each 5’ and 3’ end). The software designed the corresponding *QQS* gene-specific Gibson primers and *QQS* fragment primers (**Supplementary Table S1**) to assign to the QQS gene 5’ and 3’ overlapping regions via PCR amplification. Gibson *QQS* fragment was generated through a PCR reaction involving the synthesized *QQS* fragment primers with overlapping regions and the plasmid pCU57, comporting the optimized *QQS* gene sequence as a template. Q5 High-fidelity DNA polymerase (New England Biolabs, USA) was used for the PCR reaction following the manufacturer’s protocol at an annealing temperature of 72°C (New England Biolabs Tm calculator tools). The outcome of this PCR reaction was an amplicon of a linearized *QQS* gene comporting overlapping sequences to its 5’ and 3’ ends. The pSAT1 vector, our initial entry shuttle vector, was also linearized using a double restriction enzymatic reaction with Sac1 and Kpn1 to generate a linearized vector for the Gibson reaction. Then, the linearized *QQS* gene comporting overlapping regions, the linearized vector was purified using gel, and purity and concentration were determined. The fragments (linearized *QQS* gene and PSAT1 vector) were combined in a single Gibson reaction following NEBuilder^®^ HiFi DNA Assembly Master Mix/NEBuilder HiFi DNA Assembly Cloning Kit protocol. An aliquot from the Gibson reaction, supposed to contain the correct assembled vector pSAT1-*QQS*, as shown in the **Supplementary Materials S1** (A), was transformed by heat shock into *E. coli* 5α competent cells (New England Biolabs, USA) for assembled pSAT1-*QQS* propagation and insert verification. Colony PCR and double restriction digestion of the assembled pSAT1-QQS vector with SacI and KpnI were performed to confirm the proper insertion of the QQS-optimized gene in this vector.

#### Insertion of *QQS* expression cassette from pSAT1*-QQS* assembled vector into pPZP-RC2-*npt-II* plant transformation vector through ligation-dependent technique

**Figure 1:**
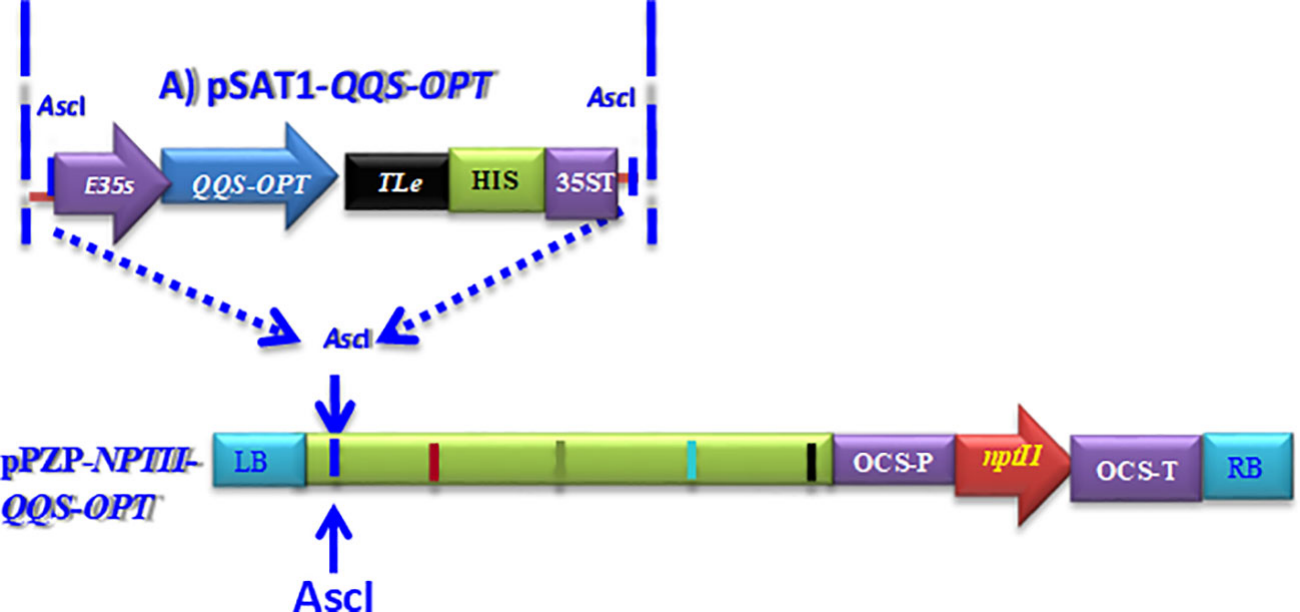
Schematic depiction of the pPZP-*QQS-NPTII-OPT* expression construct designed for the constitutive expression of optimized *QQS* in genetically modified cassava plants. Building the *QQS* expression construct involves cloning the optimized *QQS* into the pSAT1 shuttle vector, designated as pSAT1-*QQS-OPT*. The process of deriving the plant expression vector includes the use of the Ascl as a homing restriction enzyme and DNA cleavage point for targeted insertion of the *QQS* expression construct into the Ascl restriction site located within the T-DNA borders, right-border (RB), and left border (LB) of the pPZP-RC2-*nptII* binary vector to generate the pPZP-*nptII-QQS-OPT* expression vector for cassava transformation. Key elements of the expression construct include the Enhanced 35S promoter (E35S) to drive the expression of the optimized QQS in bioengineered cassava plants, the Translation Enhancer (TLE) derived from the potato etch virus, the 35S terminator sequence, and histidine tag for transgenic protein detection, *npt*II for plant selectable marker gene. Within the T-DNA borders of the pPZP-RC2-*npt*II binary vector, the *nptII* expression for plant-selectable marker selection will be controlled by the octopine synthase gene's promoter (OCS-P) and terminator (OCS-T).

pPZP-RC2-*npt-II*, an 8783 bp plasmid , is a plant expression binary vector as described by Tzfira et al. (2005); Dafny-Yelin and Tzfira (2007); Zeevi et al. (2012) as shown in (**Supplementary Figure S1C** **).** The confirmed single-gene expression cassette originating from the pSAT1-*QQS* assembled vector underwent restriction digestion with the restriction homing enzyme AscI (New England Biolabs, Ipswich, MA, USA) to enable the 1,433 bp *QQS* expression cassette to be pulled out from the vector and to facilitate its insertion into the pPZP-RC2-*npt-II* final vector. The pPZP-RC2-*npt-II* binary vector (Goderis et al., 2002) was linearized through restriction digestion with AscI homing enzyme (New England Biolabs, Ipswich, MA, USA). Following the manufacturer’s protocol, the resultant digestion products were purified using the QIAGEN MiniElute Gel Extraction Kit (QIAGEN, USA). The product of the reaction, a linearized pPZP-RC2-*npt-II*, and the *QQS* expression cassette were dephosphorylated with the shrimp alkaline phosphatase (NEB, USA) following instructions from the manufacturers. The linearized and dephosphorylated Q*QS* expression cassette and pPZP-RC2-*npt-II* vector were ligated using T4 DNA ligase (New England Biolabs, Ipswich, MA, USA). An aliquot of the ligation product was transformed into *E. coli* DH5α competent cells for assembled plasmid propagation and insert verification. Colony PCR was performed on positive colonies of *E. coli* DH5α, and five positive colonies were sequenced at Macrogen, USA, to confirm the integrity of the inserted optimized QQS sequence into pPZP-RC2-*npt-II*. In addition to colony PCR, the proper cloning of the QQS expression cassette into the pPZP-RC2-*npt*-II-*QQS* final expression vector was confirmed through restriction digestion with ASCI homing enzyme and expression cassette fragment confirmed on the 1% agarose gel electrophoresis. The correct *QQS* expression construct (pPZP-RC2-*nptII*-*QQS*) (**Figure 1**) was mobilized into disarmed *A. tumefaciens* strain LBA4404 (Lin et al., 2005) by heat shock. Colony PCR was performed on positive *A. tumefaciens strain* LBA4404 colonies to assert the conformity of the *QQS* expression construct into the disarmed *A. tumefaciens* strain LBA4404. Furthermore, *A. tumefaciens* strain LBA4404 trans-conjugant was used as a *vir* helper strain and vector system for cassava transformation (Taylor et al., 2001; Hankoua et al., 2006; Taylor N. et al., 2012; Ligaba-Osena et al., 2018).

#### Genetic transformation: Insertion of *QQS* inside cassava genome and regeneration of putative transformed cassava plant via *Agrobacterium-mediated* gene delivery

##### Production of homogeneous and highly proliferating friable embryogenic calli

The friable embryogenic callus (FEC) is routinely used as a target tissue for the genetic transformation of cassava. To induce and proliferate high-quality FEC tissues, we followed protocols described by (Taylor et al., 2001; Hankoua et al., 2006; Taylor N. et al., 2012) with limited modifications. Cassava cultivar TMS60444, a preferred genotype for gene insertion due to its performance in responding to the existing *in vitro* regeneration and genetic transformation systems of cassava (Ladino et al., 2002; Hankoua et al., 2006), was maintained as *in vitro* plantlets in a controlled growth chamber under the following conditions: 27°C, 80% relative humidity, and 16-h/8-h day/night cycles. Under these growing conditions, *in vitro*, plantlets are generated using stem segments comporting at least one axillary, apical, or both shoot bud cultured on Cassava Basic Medium with the following nutrients composition [4.43 g/L MS (Murashige and Skoog, 1962) salts with vitamins, 2% (w/v) sucrose, 2mM CuSO_4_, **Supplementary Table S2**]. According to Hankoua et al. (2006), Taylor et al. (2001) and Taylor N. et al. (2012), friable embryogenic calli were initiated from cyclic somatic embryos from cassava immature leaf lobes. Induced FEC was proliferated, cycled, and maintained as described by Taylor et al. (2001; Hankoua et al., 2006, 2012) in the MS2-P50 medium (**Supplementary Table S2**). To initiate FEC, high-quality and proliferating cyclic somatic embryos were fragmented into small pieces and incubated on GD250P [GD (Gresshoff and Doy, 1972) 2-50P, **Supplementary Table S2**] medium for FEC formation. The pure FEC was continuously selected from GD250P plates and subcultured every four weeks until homogenous and highly proliferating FEC clusters were obtained. Homogeneous and highly proliferating friable embryogenic calli (HPFEC) were continuously maintained on GD6-50P supplemented with 6% sucrose [GD (Gresshoff and Doy, 1972) 6-50P, **Supplementary Table S2**] before their utilization for genetic transformation.

##### *Agrobacterium*-mediated genetic transformation of cassava target tissue (HPFEC)

*Agrobacterium-mediated* DNA delivery and recovery of *in vitro* regenerated plantlets were performed following the cassava transformation and regeneration techniques described by Hankoua et al., 2006; Taylor N. et al., 2012; Osena Ligaba et al., 2018. *Agrobacterium tumefaciens* strain LBA4404 *vir* helper strains, and transconjugants harboring the control binary plant transformation vector pPZP-RCS2-*npt*II without the optimized *QQS* gene of interest and comporting the *npt*II gene coding for the enzyme neomycin phosphotransferase (NPT), an aminoglycoside phosphotransferase conferring resistance to antibiotics such as kanamycin, neomycin, paromomycin and used as the plant selectable marker gene, the expression cassette for driving the expression of *QQS* optimized gene in cassava tissue were used for genetic transformation. Single colonies of *Agrobacterium* transconjugants LBA4404 carrying each expression construct were cultured in a 2 mL LB medium containing appropriate antibiotics for overnight growth for 10-12 hours under shaking at 220 rpm at 28°C. Approximately 0.5–1.5 mL of these overnight *Agrobacterium* suspensions initiated 25 mL of Yeas Mold (YM) medium (composition for 1 L of media: yeast extract 3 g, malt Extract 3 g, peptone 5 g, dextrose 10 g) supplemented with appropriate antibiotics and 100 μM acetosyringone.

The *Agrobacterium* suspension cultures grew and were monitored to reach an OD_600_ of 0.5–0.75. The bacteria suspensions with optimal OD_600_ were prepared according to Hankoua et al., 2006; and Osena Ligaba et al., 2018 before the inoculation of HPFEC. This suspension was washed and induced in a GD2 50P medium containing 200 µm of acetosyringone and Pluronic F-68, a non-ionic surfactant, at a concentration of 0.03%. HPFEC obtained from GD2-50P agar media (**Supplementary Table S2**) was inoculated with 1 mL of induced bacterial suspensions. Three independent genetic transformation events were performed for each expression construct. After inoculating HPFEC with these bacteria suspensions, tissues were vacuum infiltrated for at least 15 min with two pauses every 5 min. These inoculations and infiltrations were incubated at room temperature for approximately 30 min.

Excess *Agrobacterium* suspensions were removed after inoculation steps, and coculturing of the inoculated HPFEC tissues was performed according to protocols described in Hankoua et al., 2006; Taylor N. et al., 2012; Osena Ligaba et al., 2018. Inoculated HPFEC tissues were placed on a co-culture plate (GD2- 50P+100 µM acetosyringone) and incubated at 22°C with full lighting for two days. Two days after cocultivation, the inoculated HPFEC were washed twice into GD2 50P containing 500 mg of carbenicillin and then placed on a recovery media plate (GD2 50P+500 mg/l carbenicillin) for seven days to initiate cell division. After seven days of recovery, HPFEC tissues were transferred to Stage I selection medium GD2-50P containing 27.5 μM paromomycin and 500 mg/L carbenicillin. PCR validated transgenes insertions from genomic DNA samples isolated from proliferating putative transgenic homogeneous and proliferating friable embryogenic calli.

#### Recovery of putative transgenic tissues, maturating embryos, and plantlets

**Figure 2:**
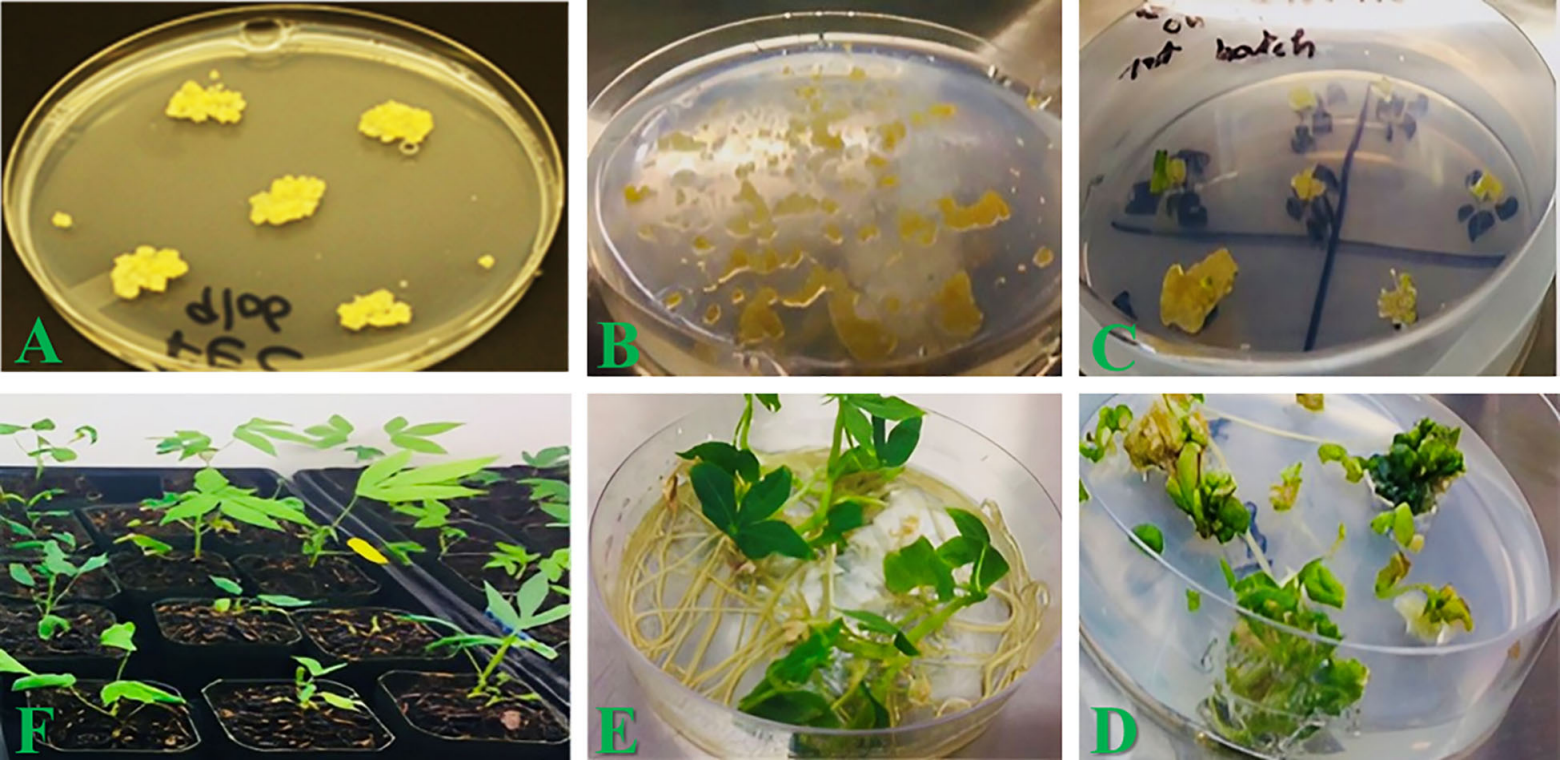
Tissue culture steps for transforming Highly Proliferating Friable Embryogenic Calli (HPFEC) of cassava, recovery, and hardening of transgenic plants. **(A)** HPFEC of cassava were generated as described in the Section "Materials and Methods." **(B)** Paromomycin selection of inoculated HPFEC with *Agrobacterium* strain LBA4404 containing an expression cassette of optimized *QQ*S gene as in **Figure 1** and production of putative transgenic calli. **(C)** Recovery of putative transgenic immature and cotyledonary stage somatic embryos selected under paromomycin pressure. **(D)** Germination and regeneration of 1st putative transgenic cotyledonary stage somatic embryos to recover putative transgenic shoots.). **(E)** Elongation and rooting of putative transgenic shoots to generate putative transgenic plantlets. **(F)** Transplantation of putative transgenic cassava plantlets into soil transgenic for acclimatization.

Putative transgenic HPFEC tissues, which are those that survived and proliferated on 27.5 µM paromomycin, were placed onto embryo maturation media (EMM) with the following composition [4.43 g/L MS salt with vitamins, 2% (w/v) sucrose, 45 µM paromomycin, 0.5 mg/L NAA, pH5.8, **Supplementary Table S1**] for the emergence of the putative transgenic embryo. Putative transformed maturing embryos were monitored from antibiotic selection media, then picked regularly, and transferred onto embryo germination media (EGM) [4.43 g/L MS salt with vitamins, 0.4 mg/L BAP, 2% (w/v) sucrose, **Supplementary Table S2**] for 3 to 4 weeks for putative transgenic shoots emergence. Germinated, putative transgenic shoots were transferred to CBM without antibiotics for rooting (**Figure 2** **).**

#### Transplantation of *in vitro* propagated transgenic and wild type plants to soil

Three-week-old micro-propagated plantlets thriving on CBM (**Supplementary Table S2**) were transplanted in Kord 3.0 square pots filled with 230 mL Fafard 52 soil (Maryland Plants & supplies INC., MD, USA), then treated with Gnatrol to control fungal gnats for hardening. This acclimatization protocol was slightly modified (Zidenga et al., 2012), which used the Fafard 51 mix to acclimate *in vitro* cassava plantlets and was shown to promote mini-tuber development. Nitrogen-Phosphate-Potassium (NPK) fertilizer and all-purpose plant fertilizer, Miracle-Gro, USA, were added, by alternation, once a week to the Fafard 52 soil for the fertilization of the greenhouse acclimatized *in vitro* plantlets (**Figure 2**) until established plants were morphologically assessed and biomass harvest for downstream characterization.

#### Molecular characterization of putative transgenic lines

Preliminary evidence of the insertion of the *QQS* gene into the genome of transgenic lines was validated through PCR reactions conducted at various stages of cassava plant regeneration and development. Biomass samples were collected from putative transgenic sources, including 1) maturing embryos, 2) three-week-old cassava plantlets cultivated on MS2 cassava maintenance medium, and 3) six-week-old soil-grown cassava plants that were well-established. The transgenic biomass derived from the six-week-old well-established soil-grown cassava plants was utilized for quantitative RT-PCR analysis to assess the expression level of the QQS gene across different transgenic lines and to conduct their biochemical characterization. Furthermore, to evaluate the expression or transcript levels of *QQS* in root and leaf tissues of various transgenic lines, total RNA was extracted from newly fully expanded leaves and roots of each selected transgenic line and wild-type non-transformed plants. The tissues were ground in liquid nitrogen using a mortar and pestle. Total RNA was extracted using Spectrum Plant Total RNA kit (Sigma, Aldrich, USA). For each transgenic line, 4 μg of the extracted RNA was treated with DNase using amplification-grade deoxyribonuclease I in a kit (Invitrogen Life Technology, USA). After DNase treatment, 16 μL of the resulting reaction was used to synthesize cDNA using superscript III first-strand cDNA synthesis system for RT-PCR (*In vitro* gen life technology, USA). The cDNA concentration was checked on Nanodrop, and then samples were normalized to 1.25 µg/μL for their use in quantitative real-time (QRT) PCR reaction. QRT-PCR *QQS* primers (**Supplementary Table S3**) were synthesized by Integrated DNA Technologies (IDT, Coralville, IA, USA). QRT-PCR was carried out using Power SYBR Green Master Mix (Applied Biosystems, USA). The Cassava tubulin gene was used as an internal control. Three technical replicates were used for each biological replicate (transgenic line), and the wild-type non-transgenic line was used as the reference sample. Relative gene expression was calculated using the ΔΔ*CT* method available on the 7500 and 7500 fast real-time PCR system version 2.0.1 software. The mean and standard error of the RQ values collected from the real-time PCR data were used to compare gene expression levels between lines.

### Biochemical characterization of wild-type and stable *QQS* expressing transgenic cassava lines

Leaves, stems, and roots biomasses were harvested from 6 weeks *in-vitro* and soil-grown of five selected *QQS* stable transgenic lines (R’’’(G) L1, R’’’(G) L2, R’’’(G) L3, R’’’ (LA) L2, R7 (F)), lines expressing only *NPT*II gene known as empty vector expressers, and wild-type plants expressing no gene for biochemical characterization. These biomasses were ground in liquid nitrogen and then stored at -80°C in 50 mL sterile centrifuge tubes. Later, these biomass samples were utilized for biochemical analysis to compare the total protein content, amino acid composition, and soluble carbohydrate content between wild-type non-transformed, empty vector expressers and stable transgenic lines expressing *QQS*.

#### Total soluble protein purification and quantification

For the total soluble protein extraction and quantification, 100 mg of ground tissues of various biomass were taken from a 50 mL sterile centrifuge previously stored at -80°C. Protein extraction was performed in triplicate for each cassava line and biomass type. Total Protein extraction was done using 1 mL of cold extraction buffer (200 mM NaCl, 1 mM Tris-HCl pH 7.8, 4% of 2- mercaptoethanol, and 1% complete protease inhibitor). After extraction of the total protein, the protein samples were vortexed and centrifuged to obtain protein pellets. These protein pellets were further suspended in 100 μL resuspension buffer, and the resulting aqueous solution was used for total protein quantification. Following the manufacturer protocol, total protein content was determined using a protein quantification CB-X kit (G Biosciences, Maryland Heights, MO, USA). The absorbance of the protein samples at 595 nm was recorded from a Thermofisher US-Vis 260 evolution spectrophotometer to determine the protein concentration (μg/μL) according to the best line of fit equation obtained by generating a standard curve using Bovine Serum Albumin (BSA) 2 mg/ml and according to the CB-X kit protocol.

#### Total soluble carbohydrate extraction and quantification

Total carbohydrate extraction and quantification were performed on ground tissues of various biomass taken from a 50 mL sterile centrifuge previously stored at -80°C. For each cassava line and tissue type, 50 mg of ground tissues were used with two replicates per sample. Soluble total carbohydrate was extracted using 200 μL of an ice-cold assay buffer supplied by the soluble total carbohydrate Assay kit (Sigma-Aldrich, USA) used for the analysis. After carbohydrate extraction, aqueous samples were centrifuged (13,000g/5min) to obtain carbohydrate pellets. These carbohydrate pellets were further re-suspended in 30 μL buffer, and the resulting aqueous solution was used for total carbohydrate quantification following the manufacturer’s protocol. Water was used for samples with less than 30 μL of pellet resuspension solution to adjust the volume to a final volume of 30 μL as required by the protocol. The absorbance of the samples at 490 nm was used to determine the soluble total carbohydrate concentration (μg/50 mg) according to an equation obtained by generating a standard curve using a D-glucose standard 2 mg/ml and according to the soluble total carbohydrate Assay kit protocol.

#### Amino-acid analysis

Amino acid analysis was performed according to the method described in Cohen and De Antonis, 1994. Briefly, oven-dry samples were hydrolyzed in 6.1 N HCl containing a small amount of phenol; the hydrolysis flasks were extensively purged of oxygen using a PicoTag workstation (Waters Corp., Milford, MA) and then incubated at 110°C for 20h. Hydrolyzed samples were then evaporated to dryness under vacuum and derivatized using AccQFluor reagent (Waters) according to the manufacturer’s directions. Chromatography was performed using procedures described as ‘System 1’ in Cohen and De Antonis (1994), with α-aminobutyric acid as an internal standard. Separation was achieved using an AccQTag C18 reverse phase column (Waters) and detection by fluorescence using excitation with 250 nm light and measured emission at 395 nm. Hydrolysis, derivatization, and analysis of each sample were performed in triplicate.

### Gene network construction and GO enrichment analysis

This analysis constructed a gene network using the Gene IDs of the At*QQS* gene in *Arabidopsis*: At*QQS* (UniProt ID- Q3E7K4). First, the Gene ID (*QQS*) was used as a query against the STRING database via Cytoscape and StringApp (Doncheva et al., 2019). STRING is a biological network database that stores known and predicted interactions obtained from primary databases and computational approaches, respectively (Szklarczyk et al., 2023). Next, we evaluated the biological role of the generated network using ClueGO/CluePedia (Bindea et al., 2009) apps in Cytoscape. Finally, a right-sided hypergeometric test (enrichment) with Bonferroni correction was used to compute the false discovery rate of each pathway and biological process to assess its significance.

### Statistical analysis

Statistical Analysis System (SAS) Enterprise Guide 5.1 was used for statistical analysis. Experiments were done in duplicates for soluble total carbohydrate content, and sampling was performed using independent biological triplicates for total protein content. One-way ANOVA was used to check differences between the mean values. Significant probability was set at p ≤ 0.05. In addition, Excel’s Microsoft version 2016 was used to check correlations between QQS transcript levels in transgenic root and leaf, the soluble total carbohydrates, and the soluble total protein content of these plant parts. Correlations analyses were also performed to determine any relationship between carbohydrate and protein content in leaves and roots.

## Results

### Phenotypic characterization of putative transgenic cassava lines and wild-type non-bioengineered plants

**Figure 3:**
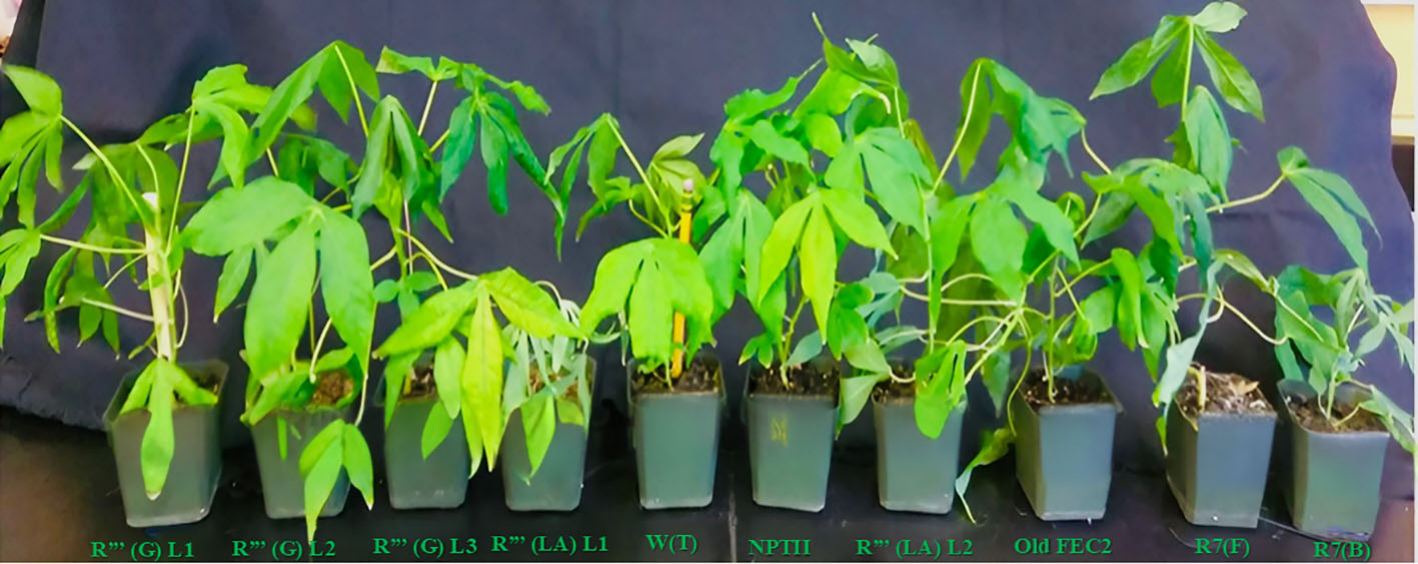
Phenotypes of soil established *in vitro* putative transgenic cassava lines expressing *QQS* gene compared to the wild type (WT) non-bioengineered and *NPT*II expressing transgenic lines. All *In vitro* plantlets showed a 100% survival rate after transplantation in soil, and transgenic plants in soil look normal with no detectable morphological anomalies compared to their non-transformed counterparts (WT) and *NPTII* (empty vector) expressers.

**Figure 4:**
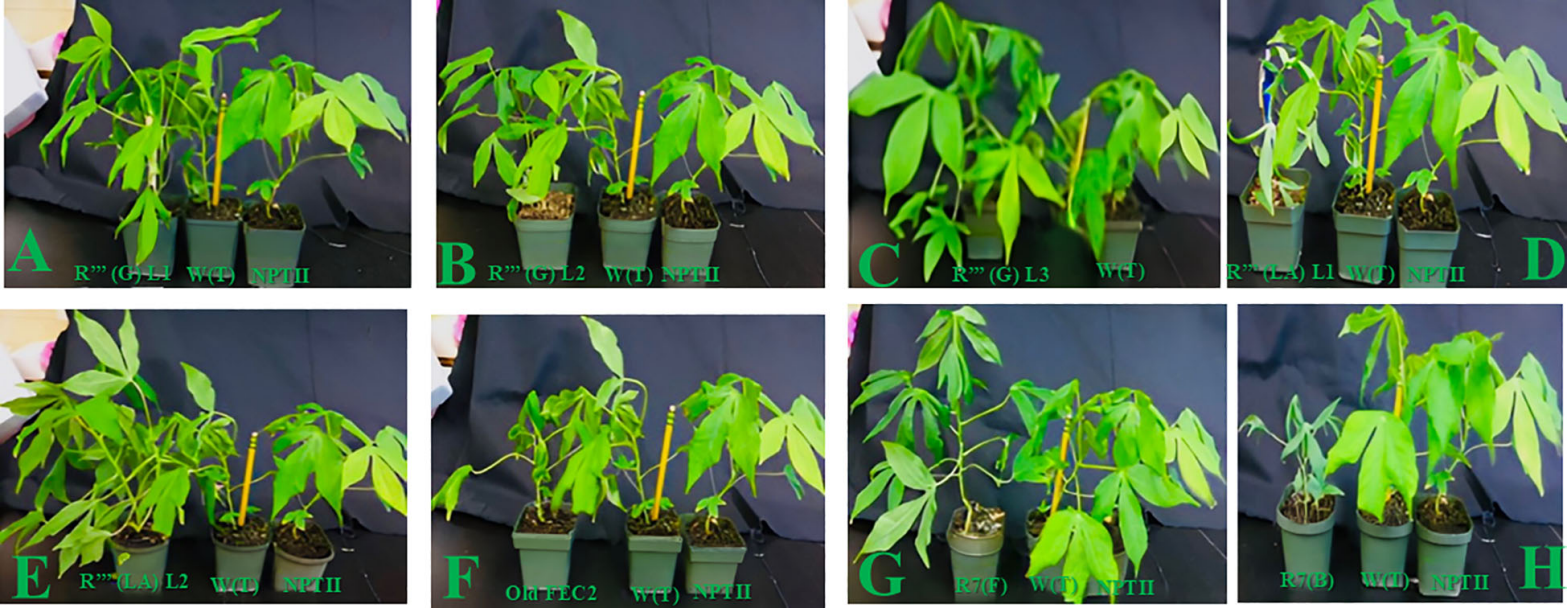
Comparative morphological analysis of 6-week-old *in vitro* putative transgenic cassava plants, established in soil, which illustrates distinctions between wild-type non-transformed plantlets and NPTД-expressing transgenic cassava, and *QQS* expressing lines R""(G)L1 **(A)**, R""(G)L2 **(B)**, R""(G)L3 **(C)**, R""(LA)L1 **(D)**, R""(LA)L2 **(E)**, Old FEC **(F)**, R7(F) **(G)**, and R7(B) **(H)**.

**Figure 5:**
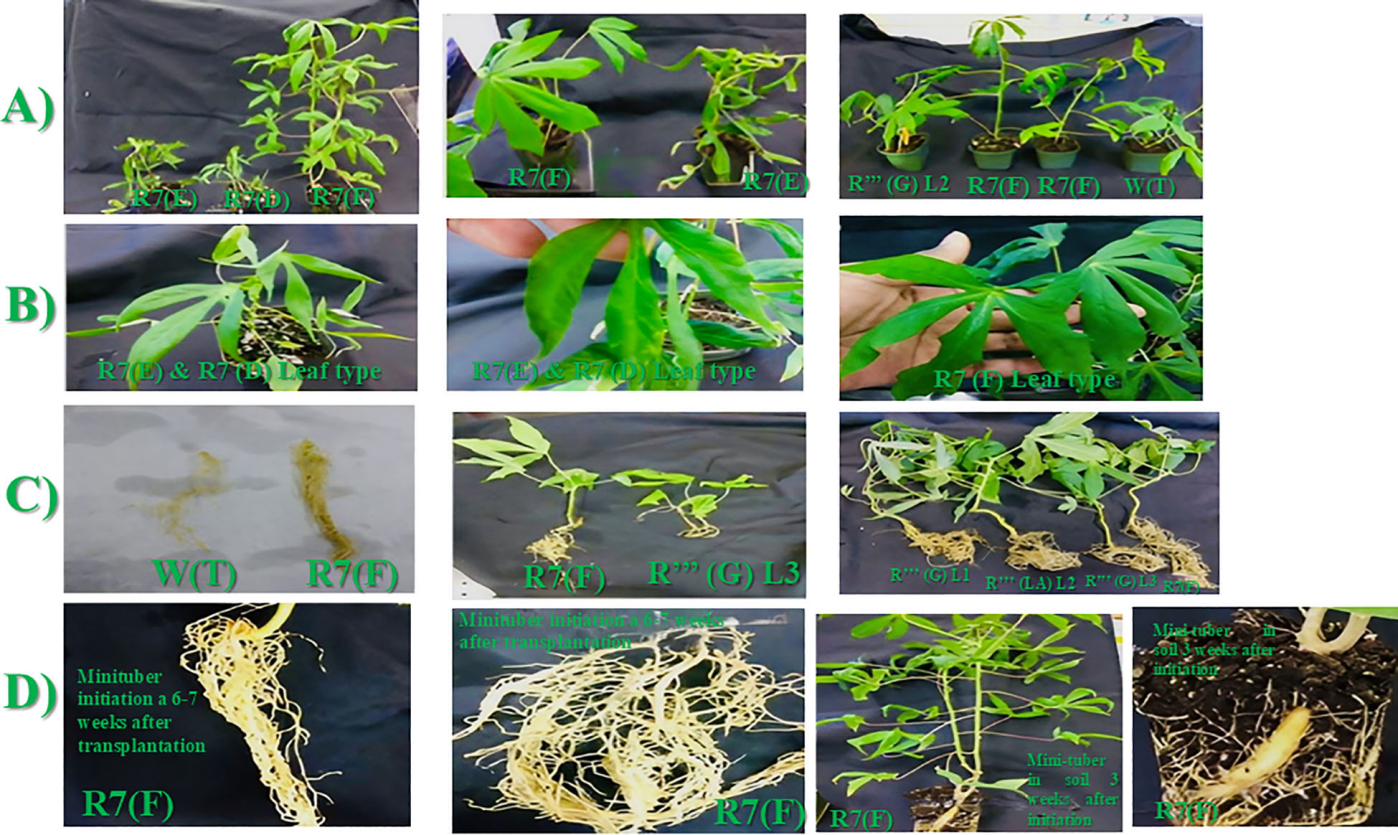
**(A-D)**: QQS expressing transgenic cassava line R7(F) exhibits a remarkable ability to make more biomasses, including larger leaves **(A, B)** and root system **(C)** and the ability to initiate mini tubers faster in- vitro **(D)** (6-7 weeks) and in soil (3-4 weeks) compared to other *QQS* expressers and WT non-transformed cassava.

Overall, 16 independent putative transgenic *in vitro* cassava plants were recovered from all *Agrobacterium*-co-cultures and plant recovery. Seven of these transgenic plants were lost due to recurrent *in vitro* microbial contaminations. *In vitro*, putative transgenic plantlets at the onset of soil transplantation look normal with no detectable morphological anomalies as compared with their non-transformed counterparts (WT) and *NPTII* (empty vector) expressers (**Figure 3** **).** All *In vitro* putative transgenic plantlets showed a 100% survival rate after transplanting in soil (**Figure 3** **).** Visual comparison of plant height and biomass production at 6-7 weeks after soil transplanting between wild-type, *NPTII*, and each transgenic cassava is shown in **Figure 4**. No marked variation in these agronomic parameters was observed between these lines, except that line R7 (B) showed slow growth with lower biomass production than the wild type and *NPTII* expresser (**Figure 4**). Symptoms of fascinated leaf, tiny stem, and slow growth were observed in two soil-established cassava expressing *QQS* lines (R7 (E), R7 (D) (**Figure 5** Section A and Section B). Among all these transgenic cassava lines, line R7 (F) exhibited a particular phenotype in its growth habit and capability to produce more biomass (**Figure 5A**). It is evident in **Figure 5A** that Line R7 (F) grows more profusely and produces more biomass than the other transgenic lines, such as R’’’ (G) L2 and the WT. Furthermore, morphological differences between individual plants of line R7, including variation in growth rate, leaf shape, and size, were observed (**Figure 5**).

Moreover, **Figure 5** section C shows the disparities in plant height, leaf production, and root size of these transgenic cassava lines. Line R7 (F) grows taller and produces more shoot and root biomass than line R’’’ (G) L3 and wild-type non-transformed plants. Similarly, line R7 (F) plant size and root size were compared with the ones obtained with lines R’’’ (LA) L2, R’’’ (G) L3, and R’’’ (G) L1. These morphological data demonstrate a higher and unique growth ability for line R7 (F) compared to all other transgenic lines and control non-transformed plants. All regenerated plants were transferred from *In vitro* culture to soil under the same growing conditions and evaluated at the same age (6-7 weeks). The final section, D in **Figure 5**, illustrates the higher capability of R7 (F) to induce mini tubers quicker after its *In vitro* plantlets were transferred to the soil between 6-7 weeks compared with other transgenic lines and wild-type plants, which did not exhibit this early tuberization capability after more weeks of growth in soil.

### Molecular analysis of putative transgenic cassava lines

#### Evaluation of *QQS* gene expression by quantitative RT-PCR

**Figure 6:**
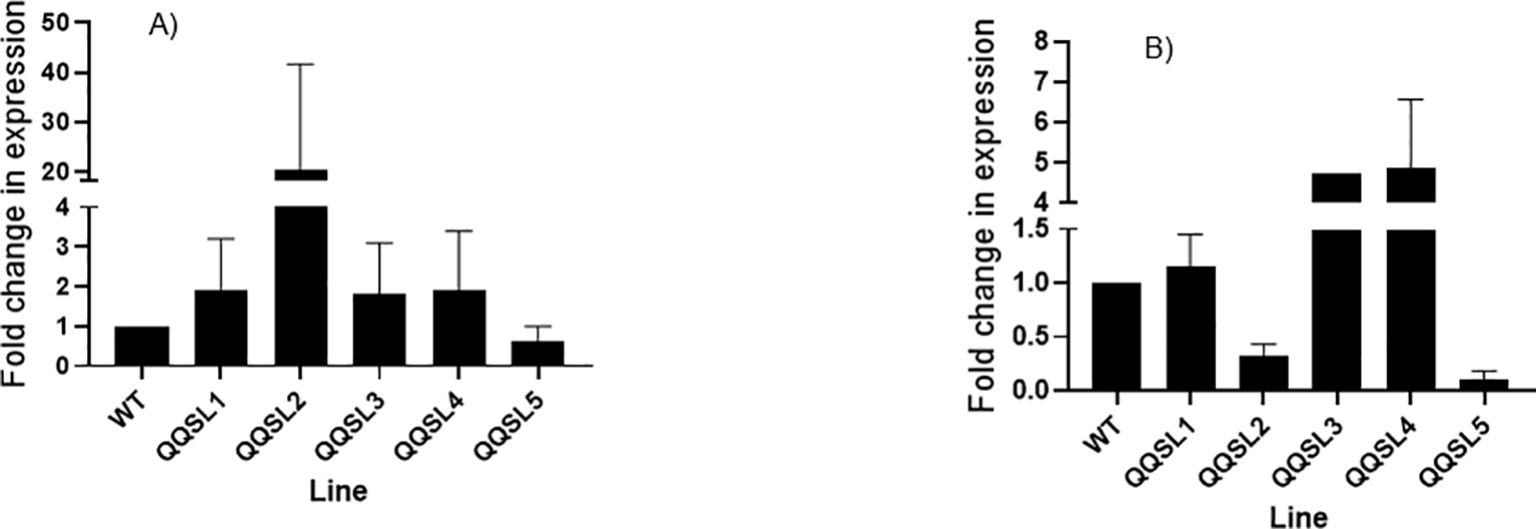
Quantitative real-time PCR analysis of QQS gene from leaf **(A)** and root tissues **(B)** of six-week- old soil-grown putative transgenic cassava plants. The graphs were plotted using the mean RQ of samples from each line and the standard error (SE) obtained after the real-time PCR. QQSL1: R"" (G) L1; QQSL2: R"" (G) L2; QQSL3: R”” (G) L3; QQSL4: R”” (LA) L1; QQSL5: R7 (F); **(A)** *QQS* transcript expression in different transgenic lines in roots, **(B)** *QQS* expression in different transgenic lines in cassava leaves. The samples were measured in triplicates. Data represent means Standard deviation; n=3 biological replicates. Error bars show the standard deviation from the mean. One-way ANOVA determined statistically significant differences from WT plants.

Genomic DNA was extracted from eleven randomly selected putative transgenic lines. Insertion of the *NPTII* and *QQS* was validated (data not shown) using the genomic DNA and gene-specific PCR primers for *QQS* (230 bp) and the plant selectable marker (*NPTII*, 790 bp) (**Supplementary Table S1** **).** The level of *QQS* gene expression in the leaf and root biomass of the wild-type and five putative transgenic lines was performed using SYBR green master mix and qRT-PCR primers (**Supplementary Table S2**), where the QQS was used as a target gene, and the *Manihot esculenta* Crantz) *α-tubulin* gene was used as an internal control. The results obtained from the expression analysis of *QQS* in leaves and roots are shown in **Figure 6**. The data showed that the *QQS* gene is differentially expressed in the leaf and root tissues of all the transgenic lines tested. Transcript levels varied widely between lines and tissue types. Furthermore, the putative transgenic line QQSL2 produced the highest transcript level for the leaf. In contrast, its transcript abundance was lower in the roots than in the other transgenic lines and the non-transgenic control. *QQS* expression in roots of lines QQSL3 and QQS L4 was higher than the non-transgenic control and other transgenic lines. In line QQSL5, the *QQS* transcript level was much lower than the other lines in leaf and root tissues. Moreover, the expression of QQS showed a very sharp decrease in the root compared to the leaf tissue in cassava line QQSL2. For root tissue, lines QQSL5 and QQSL2 generated the lowest level of QQS transcript, even below the transcript of the reference wild-type non-transformed plants.

### Biochemical analysis of stable transgenic cassava plants

Biochemical analyses were performed on a wild-type non-transformed cassava line and an NPTII-expressing line, and five confirmed transgenic lines were named R’’’ (G) L1, R’’’ (G) L2, R’’’ (G) L3, R’’’ (LA) L2, and R7 (F). Positive quantitative Real-time PCR data in **Figure 6** demonstrated that the QQS gene is well integrated into the genome of these transgenic lines. This positive data prompted us to select these lines for further biochemical analyses.

#### Total soluble protein content from leaves, stems, and roots biomass of stably transformed cassava transgenic lines

**Figure 7:**
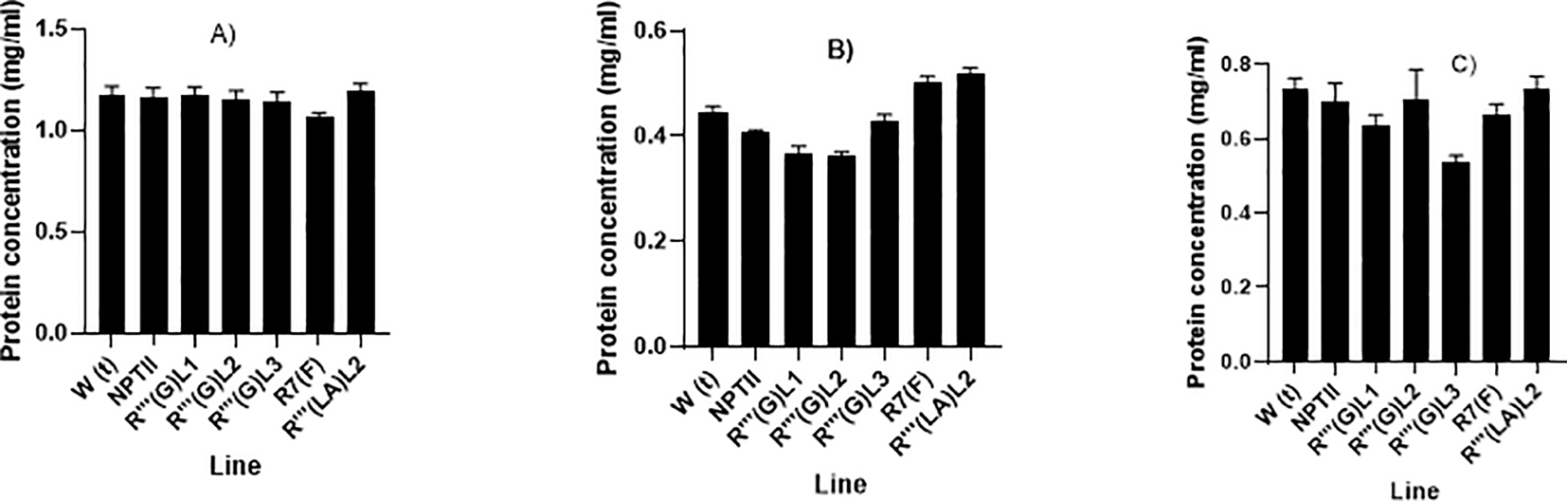
**(A-C)**: Quantitative evaluation of total soluble protein concentration in leaves **(A)**, stem **(B)** and root **(C)** tissues of six weeks *in-vitro* soil grown transgenic cassava plants. The graphs were plotted using the mean protein concentration of samples from each line and the standard error (SE) of the mean of three replicates.

Biochemical analyses, including protein and soluble carbohydrate contents, were performed on wild-type non-transformed cassava line, *NPTII* expressing line, and five transgenic lines R’’’ (G) L1, R’’’ (G) L2, R’’’ (G) L3, R’’’ (LA) L2 and R7 (F). Total soluble protein was analyzed using the Coomassie Bradford protein assay kit following the manufacturer’s procedure using BSA as the standard (Thermi Scientific). **Figure 7** shows that the samples’ soluble total protein concentration varied depending on the tissue types analyzed. All the stable transgenic lines and the two control non-*QQS* expressers (WT and *NPTII*) make more protein in the lead and stem biomass, with root biomass making less protein for all the lines. For leaf tissues, no significant differences were observed in total protein concentration between all the putative transgenic lines except biomass of line R7 (F), showing a slight reduction in soluble total protein concentration (1.1µg/µL) compared to other lines and the two controls non-*QQS* expressers (1.3 µg/µL for WT and *NPTII*). More variabilities in the total protein concentration were observed for stem and root biomass, with lines R’’’ (G) L1 (0.6 µg/µL) and R’’’ (G) L3 (0.5µg/µL) showing a significant protein decrease in total protein concentration compared to other lines, especially WT (0.75 µg/µL) and *NPTII* (0.70 µg/µL) biomass. Stem biomass from other lines exhibits total protein levels comparable to control plants. Variabilities in protein concentration of root biomass were observed in all the lines, with lines R’’’ (G) (LA) L2 (0.53 µg/µL) and R7 (F) (0.5µg/µL) showing a higher protein concentration compared to other lines, especially the WT non-transformed (0.42 µg/µL) and the NPTII expressing line (0.40 µg/µL). Root biomass from lines R’’’ (G) L1 (0.35 µg/µl) and R’’’ (G) L2 (0.55µg/µL) showed lower protein concentration.

#### Amino-acid composition in the leaves, stems, and roots biomass of *QQS* stably transformed cassava plants, non-*QQS* expressers (WT and *NPTII*)

**Figure 8:**
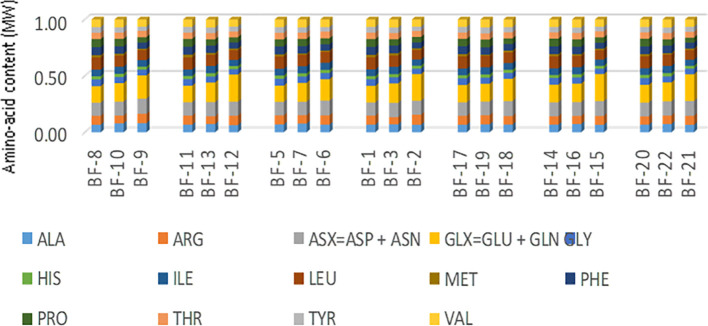
Amino acid compositions of biomass harvested from the leaves, stem, and roots of stably transformed cassava plants. It provides a summary or description of the data related to the distribution and abundance of various amino acids in the different plant parts (leaves, stem, and roots) of stably transgenic cassava plants. For each amino acid, the mean value of each sample was plotted to obtain the figure above. BF-1 :R""(LA)L2 Leaves; BF-4:R""(G)L3 Leaves; BF-5:NPTII Leaves; BF-8:W(t) Leaves; BF-11: Pure FEC Leaves; BF-14: R""(G)L1 Leaves; BF-17: R7 (F) Leaves; BF-20: R""(LA)L2 Leaves; BF-3:R""(LA)L2 Roots; BF-7:NPTII Roots; BF-10:W(t) Roots; BF-13: Pure FEC Roots; BF-16:R""(G)L1 Roots; BF-19: R7 (F) Roots; BF-22:R""(G) L2 Roots; BF-2: R""(LA)L2 Stem; BF-6:NPTII Stem; BF-9: W(t) Stem; BF-12: Pure FEC Stem; BF-15: R""(G)L1 Stem; BF-18: R7(F)Stem; BF-21: R""(G)L2 Stem;.

**Figure 8** depicts the amino acid composition data gathered from biomass samples obtained from leaves, stems, and roots of stably transformed cassava plants. Across all samples, a total of 18 amino acids were identified. The data summary shown in **Figure 8** is displaced in (**Supplementary Table S4**). Data analysis suggests no significant variation in the levels of individual amino acids between the leaves, stems, and roots biomass of stably transformed cassava plants compared to two control groups, namely non-*QQS* expressers (WT and *NPTII*). Glycine, glutamic acid, aspartic acid, asparagine, and leucine are abundant across all lines and plant parts, with glycine and glutamic acid particularly abundant in stem biomass across all lines. Conversely, histidine and methionine are the least abundant amino acids in all lines, with methionine showing a relatively higher abundance in root biomass across all tested transgenic lines. Notably, cysteine, an essential amino acid, was undetected in the analyzed samples. However, it is plausible that cysteine may be present but undetected due to the technical limitations of the employed methodology.

#### Soluble total carbohydrate composition from leaves and roots biomasses of stably transformed cassava plants expressing *QQS*, non-*QQS* expressers (WT and *NPTII*)

**Figure 9:**
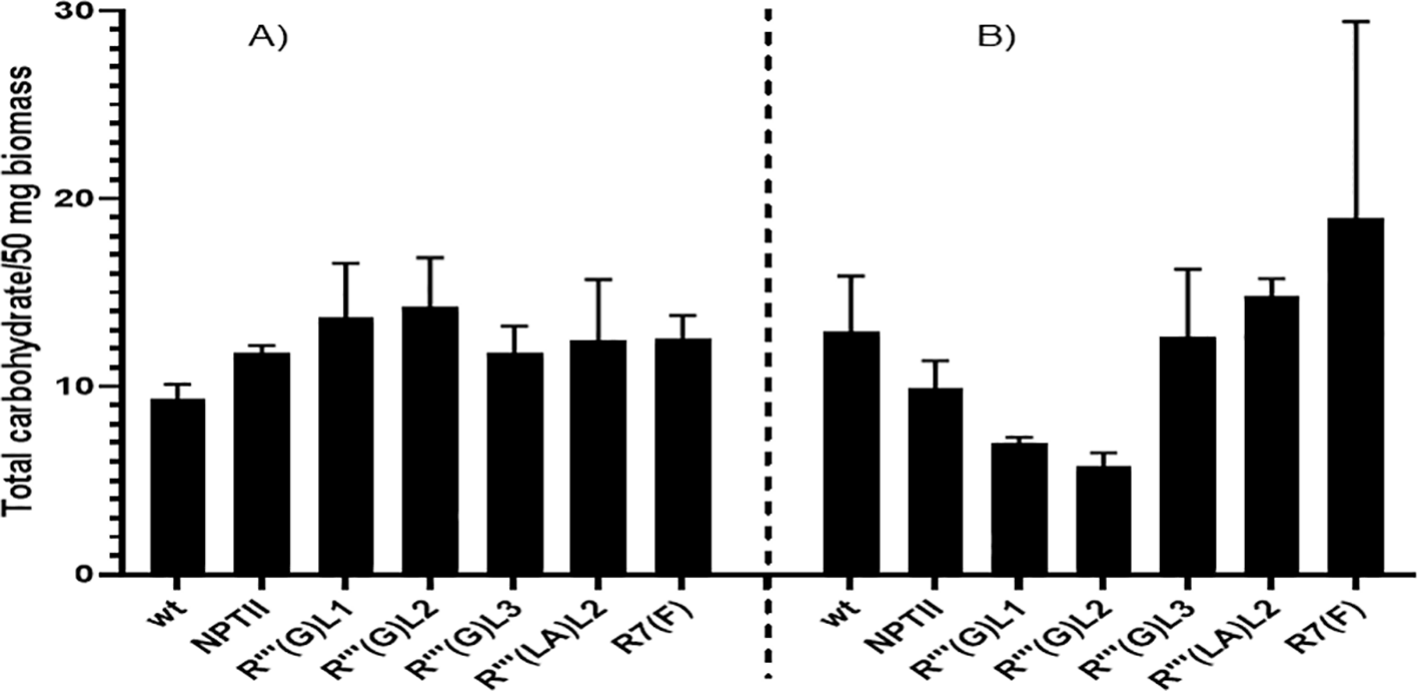
Quantitative evaluation of soluble total carbohydrate in leaves **(A)** and root tissues of wild-type, NPTII, and QQS expressing lines **(B)** of six weeks in vitro soil- grown cassava plants. The graphs were plotted using the mean total carbohydrate content of samples from each line and the standard error (SE) obtained after carbohydrate analysis. The samples were measured in triplicates. Data represents the standard error of two biological replicates. Bars represent the means and standard error of at least two independent experiments.

The total soluble carbohydrate is determined by a kit purchased from Sigma according to the recommended procedure. **Figure 9** shows large leaf and root carbohydrate concentration variabilities for all the lines analyzed. In leaf biomass, line R’’’(G)L1 and R’’’(G) L2 produced the highest soluble total carbohydrate content, reaching approximately 13µg/50mg compared to the control non-*QQS* expressers WT (9µg/50mg) and NPTII (11 µg/50 mg). For root sample, line R’’’(G) L1 (6 µg/50mg) and R’’’(G) L2 (5µg/50mg) produced the lowest carbohydrate content reaching approximately 13µg/50mg, and R7 (F) the highest (18µg/50mg) compared to the controls non-*QQS* expressers WT (11µg/50mg) and *NPTII* (10 µg/50mg). The root biomass of line R(7)(F) produces the highest carbohydrate content among all biomasses and lines tested.

### Correlation analysis between transcript expression levels and carbohydrate or protein content

**Figure 10:**
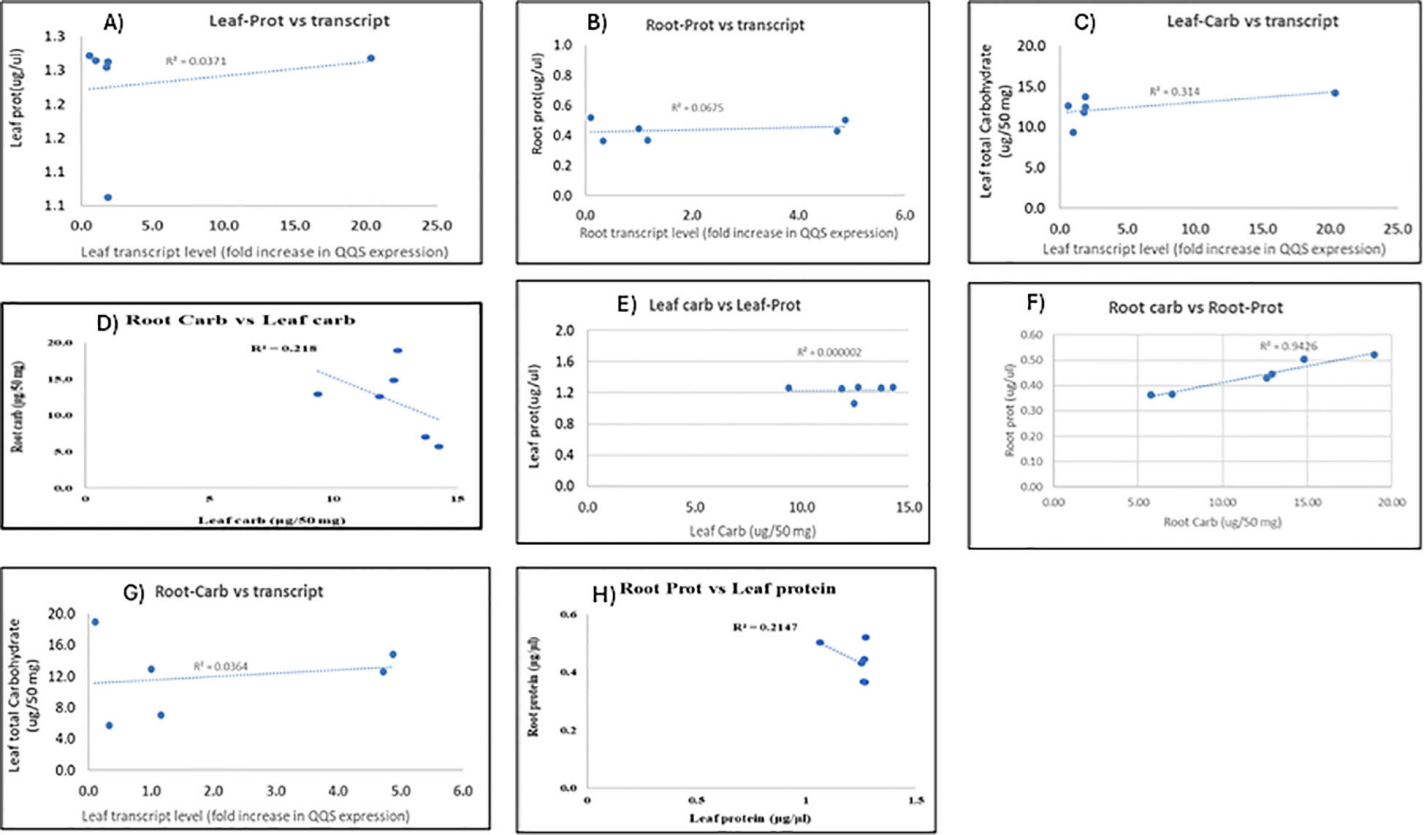
The correlation analysis between transcript expression level and carbohydrate or protein content, between carbohydrate and protein content of various plant parts (roots and leaves) of stably transformed cassava lines. The correlations between leaf; root protein content, and transcript level are depicted in Panels **(A–C, G)** show the correlations between leaf, root carbohydrate content, and transcript level. Panels **(D–F, H)** showed correlations between root and leaf carbohydrate content, leaf carbohydrate and leaf protein content, root carbohydrate and root protein content, and root and leaf protein content, respectively.

**Figure 10** shows the relationship results between *QQS* transcript levels in leaf and root tissues and their corresponding soluble total carbohydrate or protein content (**Figures 10A, B, C, G**) as well as the relationship between carbohydrate and protein content between and within leaf and root biomass (D, E, F, H) for all transgenic lines tested. This correlation analysis refers to statistical relationships between the expression levels of *QQS* transcripts and the amounts of carbohydrates or proteins in a sample. These correlations can provide insights into how gene expression might influence the production or regulation of carbohydrates and proteins within a biological system. Based on the various equations of best fit and the value of the coefficient of determination R^2^ of all the correlation data, it is evident that no strong correlation is observed between the protein and carbohydrate content of the leaf and root biomasses and the level of *QQS* transcripts produced from these plant parts in all the lines. No relationship was also depicted between the leaf carbohydrate and leaf protein for all the lines. The only positive correlation observed was between root carbohydrates and root protein. A very poor correlation was observed between the content of leaf carbohydrate and root carbohydrate, as well as the content of leaf protein and root protein.

### Gene network construction and GO enrichment analysis

**Figure 11:**
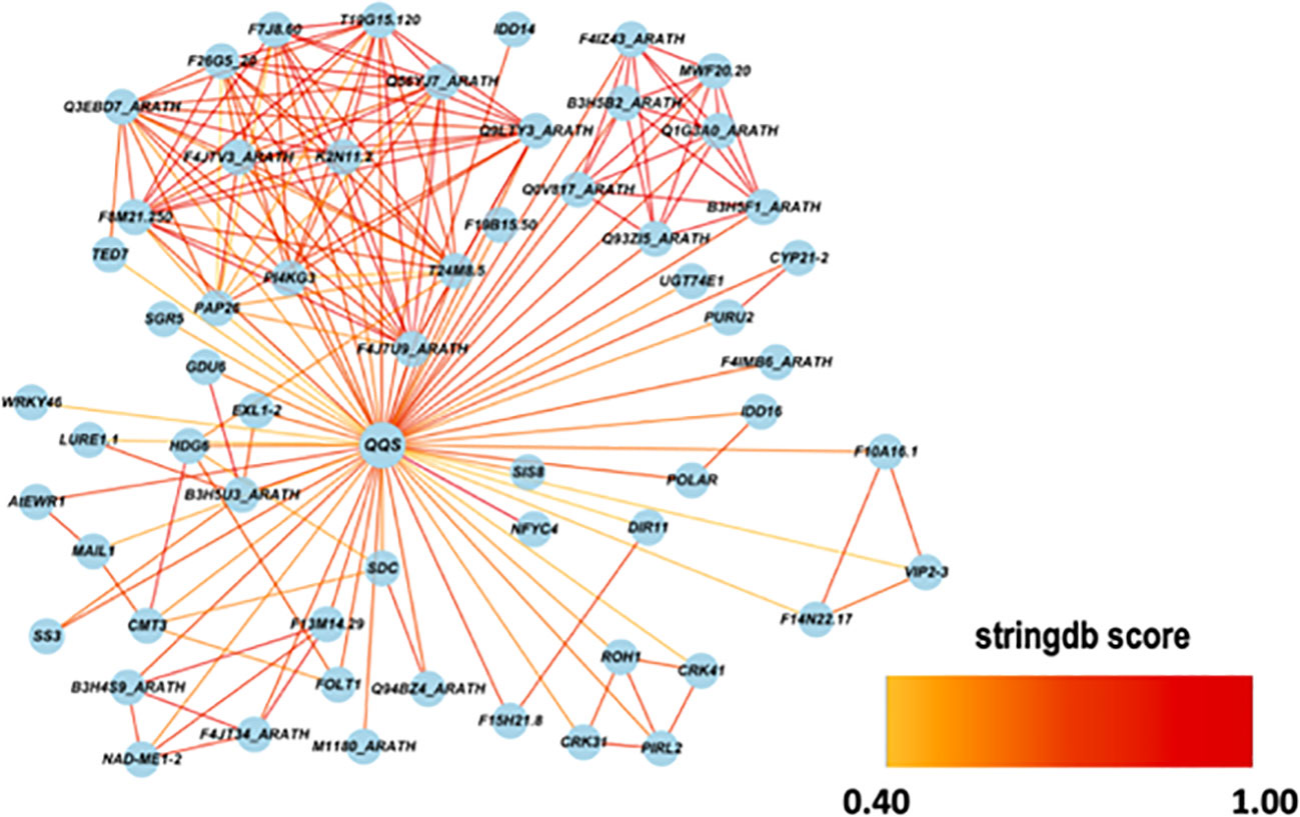
A gene network between *AtQQS* with 59 related genes and 184 interactions were obtained from the STRING database. The selected string score is 0.40 and above.

**Figure 12:**
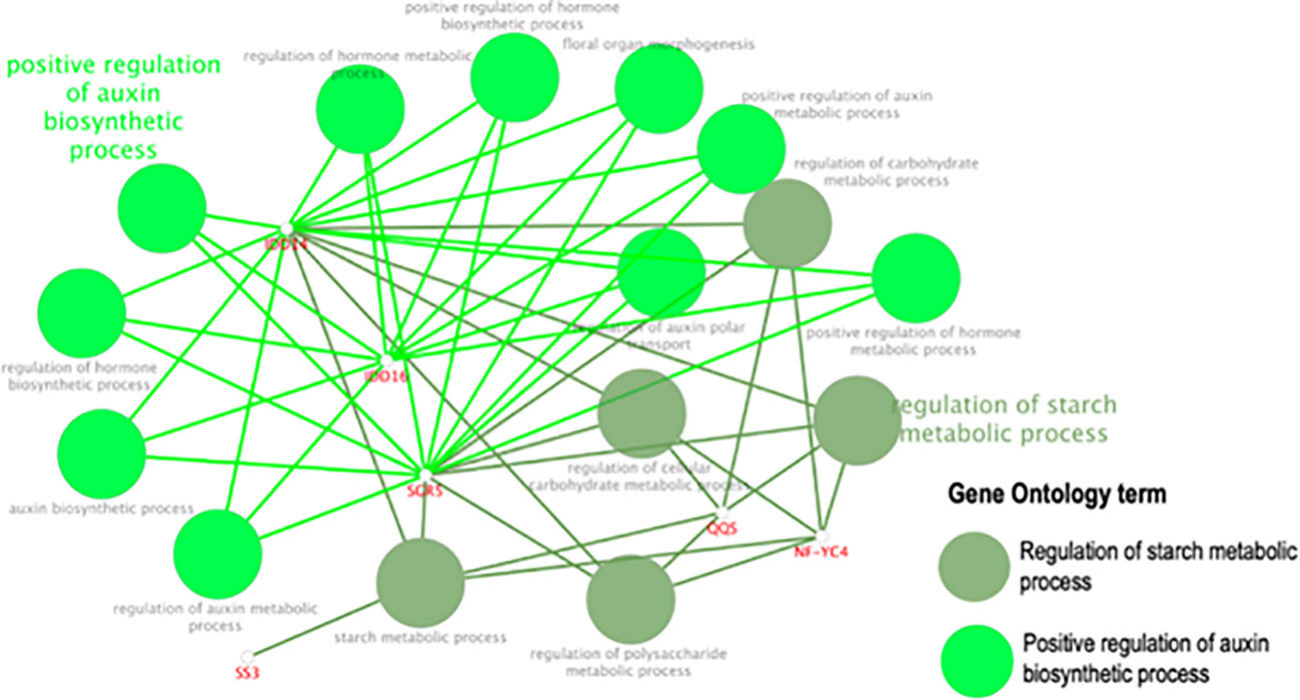
The GO enrichment using ClueGO/CluePedia apps from Cytoscape. The related genes are highlighted **Supplementary Table S7** according to the biological processes involved.

Using the STRING database, *AtQQS* was used as a bait gene to establish gene interaction. **Figure 11** shows the interaction between the query gene with 59 interacting partners, generating 184 interactions or edges. The gene network consisted of several functional categories related to the regulation of the starch metabolic process and the regulation of the auxin biosynthetic process analyzed by ClueGO/CluePedia (**Figure 12**). Detailed information on the genes and biological processes are listed in (**Supplementary Table S5**).

### Statistical analysis of correlations between variables

SAS Enterprise Guide 5.1 was used for statistical analysis. Lines were used as the independent variable, and protein or carbohydrate content in leaf, root, and stem were used as the dependent variables, with a significant level of 5%.

## Discussion

In this study, we assembled the expression cassette of optimized *QQS* sequence into the plant transformation binary vector pPZP-RCS2-ocs-nptII (Goderis et al., 2002), one of the modular plant transformation vectors that allow flexible insertion of multiple expression units was achieved through the combination of ligation-free (Gibson et al., 2009) and ligation-dependent cloning system. Out of the remaining eight stably transgenic lines, only 5 were selected for molecular and biochemical analysis because root, leaf, and stem biomasses were available. Morphological characterization of the established and soil-grown transgenic cassava lines identified four independent lines (R7(D), R7 (B), R’’’ (LA)L1, R7 (E) out of the eight successfully regenerated transgenic lines (50%) presented some form of morphological abnormalities, but R7 (B), R7 (E) showed a pronounced abnormality characterized with tiny and fasciated leaves, very tiny stem, and an overall slow growth rate. Because of these physiological abnormalities, lines R7 (B) and R7 (E) were not used for the downstream biochemical analysis because of their inability to produce enough biomass from all plant parts. The low growth rate can be related to low photosynthetic capability due to the small leaf surface area of the malformed plants. The cassava target tissues, friable embryogenic callus (FEC) used for inserting *QQS* optimized sequence into cassava genome, were 24 months old and were established and maintained from organized embryogenic tissues (Taylor et al., 2001; Hankoua et al., 2006; Taylor N. et al., 2012). The observed growth abnormality of these malformed lines could be due to the age of the FEC used in this study, which was about twenty-four months old. This observation is consistent with Taylor N. et al. (2012) *, who suggested that transformation of FEC at an optimum stage (about nine weeks after induction from organized embryogenic tissue)* and timely subculturing of transgenic tissues could reduce the formation of abnormal plants. These abnormalities could also be due to the adverse physiological and morphological effects of the recombinant *QQS* protein in transgenic cassava plants. The observed growth defect could be genetic or epigenetic variation (Machczyńska et al., 2015). The growth variability may also be due to the high-level expression of the optimized *QQS* gene, but this is contrary to the observation that the expression of *QQS* in soybean (Li L. et al., 2015) has no impact on the morphology of the field-grown transgenic soybean. However, 69.2% of the regenerated line presented enhanced morphological characteristics compared to their wild-type sibling plants with large leaves, very stick stems, high biomass production, and an early mini-tuber induction, particularly for line R7 (F) at 6-7 weeks after its *in vitro* plantlets were transferred in soil. In contrast to the observed outcomes in *Arabidopsis thaliana* and soybean, where the introduction of *QQS* expression resulted in regenerated lines that exhibited no discernible differences in morphology compared to their wild-type counterparts throughout their growth stages (Li et al., 2009; Li L. et al., 2015), the case of *QQS* expression in cassava plants, exemplified by line R7 (F), showcases a notable departure. Here, there is a notable enhancement in the physiological attributes of the plant, leading to an overall increase in plant vigor, a novel characteristic that may be attributed to the expression of *QQS* in cassava.

Data from the gene network analysis between *AtQQS* and its interacting partners generated by the STRING database and the GO enrichment using ClueGO/CluePedia showed that the gene network revealed several functional categories with *AtQQS* association to some transcription factors such as Zinc finger protein SHOOT GRAVITROPISM 5 (SGR5), a member of the SGR gene family known as regulating starch metabolism by supporting cellular gravitropism, a physiological process known to be very crucial for plant growth and crop productivity (Cho et al., 2024), and the protein indeterminate-domain 16 (IDD-16), a transcription factor regulating lateral organ morphogenesis and gravitropic responses as well as involved in the establishment of auxin gradients through the regulation of auxin biosynthesis and transport (Cui et al., 2013). There is a possibility that the expression of *QQS* in putative-transformed cassava line R7 (F) might positively regulate the expression of SGR5 and IDD-16 or their orthologues in cassava, enabling novel *QQS* expressing cassava lines to display excellent plant vigor. However, further detailed genomic studies such as global transcriptome on transgenic phenotype such as line R7 (F) will provide molecular and physiological cues on the nature of these genetic relationships, among others, between *QQS*, SGR5, and IDD-16. While biomass traits such as plant height, root, stem, and leaf biomass production are quantitative traits controlled by many genes, studies aimed at uncovering the molecular mechanism behind such traits discovered many regulators, such as transcription factors, which positively or negatively regulate the expression of these quantitative traits. Identifying such regulators (negative or positive) opened many avenues for targeting heterologous expressions or knockouts aimed to enhance the expression of quantitative traits. Studies recently showed that the overexpression of a single *Dof* transcription factor COG1 in *Arabidopsis* is a critical regulator of plant biomass by promoting photosynthesis and starch accumulation (Wei et al., 2023).

Initially identified as an orphan gene in *Arabidopsis*, *QQS* was initially characterized as a transcription factor that modulates metabolite allocation in soybean seeds (Li L. et al., 2015). The transgenic introduction of this transcription factor into the cassava genome, a crop known for its high starch production compared to the high protein content of soybean, may reveal additional interactions of *QQS* with other transcription factors, such as microRNA, known to play crucial roles in plant development, potentially resulting in enhanced biomass production (Zhang et al., 2015; Raza et al., 2023). Insertion of *QQS* and *NPTII* genes in the cassava genome was confirmed through gene-specific PCR using genomic DNA isolated from various tissues of different independent putative transgenic lines at an early stage of regeneration (3 months after transformation). Real-time PCR results revealed differentially expressed *QQS* between different lines first, within and between leaves and roots of these lines. The variability in the transcript level of a *QQS* sequence may be due to the combination of various critical genetic factors shown intensively to affect transcript levels, such as tissue type, promoter sequence, enhancer and codon usage, and transgene insertion location within the chromosome (Streatfield, 2007). We used a powerful constitutive promoter, the enhanced 35S promoter (Kay et al., 1987; Zhou et al., 2013), to drive *QQS* expression in all tissues of these putatively transformed lines and *QQS* expressing lines, but many studies have demonstrated that significant variability in the transcript levels of a transgene in various tissues is achievable even with a relatively less constitutive solid promoter and this could be due to transgenic position effect (Liu, 2013) and the physiological environment of the cell and tissue types where transgene is expressed. This position effect could explain why higher *QQS* expression was noticed in leaves, especially for the R’’’ (G) L2 line compared with other transgenic lines and the wild-type control. Furthermore, *QQS* expression in R’’’ (LA) L2 was low in leaves while very high in roots. In addition, *QQS* is highly expressed in R’’’ (G) L2 leaves while very low in the roots. Moreover, *QQS* was expressed at a low level in R7(F) both in leaves and roots, and the transgene position effect could account for these observed variabilities in *QQS* expression.

Biochemical analysis of stably *QQS* expressing lines revealed either an increase or a decrease in protein content depending on lines and a general increase in leaf carbohydrates. In contrast, a decrease in root carbohydrate was recorded for R’’’(G) L1 and R’’’ (G) L2 putative transgenic lines. In general, four tendencies have been identified in the biochemical results. For some cases where the *QQS* was expressed at a low level in leaves (R7 (F)), protein content in leaves decreased compared to the wild-type non-transgenic, while soluble total carbohydrate content was very high. This tendency of plants expressing *QQS* to decrease protein content while increasing carbohydrate production when expressed at a deficient level was also found in some transgenic *Arabidopsis thaliana* and soybean lines expressing *QQS* (Li et al., 2009). In contrast, a high expression of *QQS* in the leaves of the line (R(G) L2) reveals no variation in the protein content compared to the wild type, while carbohydrate accumulation in this line (R(G) L2 increased considerably. Li et al. (2009) demonstrated that a high expression level of *QQS* in transgenic soybean led to an increased protein accumulation while carbohydrate accumulation was decreased. This observation does not align with the observed effect of high expression of *QQS* in cassava leaves of line (R (G) L2), possibly pointing to another molecular mechanism of this transcription factor in plant development in a related soybean plant, cassava. Moreover, in cassava roots, a shallow expression of *QQS* in line R7 (F) led to the modulation of protein and soluble total carbohydrates, increasing their content. In contrast, a very high *QQS* expression in stably transformed cassava line (R (LA) L2) revealed a high accumulation of protein and soluble total carbohydrates. However, a high expression of *QQS* in R’’’(G) L3 roots revealed no noticeable variation in protein and soluble total carbohydrate accumulation. No correlations were observed between the *QQS* transcript levels, carbohydrate, and protein content for leaf and root biomasses of all the transgenic lines. This lack of relationship demonstrates that the level of *QQS* transcripts in a specific plant part of an independent and stable transgenic line does not influence the ability of *QQS* protein to exert the ability to partition the allocation of metabolites during plant development. This phenomenon was also observed in soybean lines expressing *QQS* (Li L. et al., 2015). It was also demonstrated that the effect of transcription factors such as *QQS* on the starch and protein accumulation in lines from the same transformation event may vary as the expression level of the transgene in different lines of the same transformation event may be different (Shou et al., 2004). *QQS* is a regulatory protein, and it is not unusual for regulatory proteins to have shallow expression levels (Nagaraj et al., 2011). Thus, this could be explained by the fact that only a minimal concentration of *QQS* saturates the *QQS* receptor, and any increases over this specific concentration or level do not affect their ability to impact protein and starch contents in a specific QQS expressing lines through the ability of this transgenic *QQS* protein to exert metabolic partitioning. No correlation was found between the content of leaf carbohydrate and leaf protein, leaf carbohydrate and root protein, leaf carbohydrate and root carbohydrate, but root carbohydrate and root protein content were found to be highly correlated (R²=O.9). It will be interesting to see if this ability to accumulate starch and protein in young roots is translated to mature tuberous root which is known to accumulate high starch and low protein (Chavarriaga-Aguirre et al., 2016). The lack of a linear relationship between the content of carbohydrates and protein from leaf, root, and stem biomass of transgenic lines versus *QQS* transcript was also observed in *QQS* transcript accumulation in *Arabidopsis* and soybean (Li L. et al., 2015). However, in the case of *Arabidopsis* and soybean, the elevation of *QQS* RNA accumulation was related to an increase in protein accumulation and a decrease in starch accumulation (Li L. et al., 2015), therefore validating one of the unveiled molecular mechanisms of *QQS* (Li et al., 2009). For cassava, line R”‘ (G) L1 and line R”‘(G) L2 leaf protein did not increase significantly compared to the control plants but have significantly reduced carbohydrate content, and this could be attributed to the *QQS* expression and modulation effect in metabolites distribution as demonstrated in *Arabidopsis* and soybean *QQS* expressers. Transgenic cassava line R7(F) produced the lowest level of *QQS* transcript in both root and leave tissues but produced the highest soluble protein increase in the root tissue compared to all the other root and leaf samples for all the lines tested. This phenomenon was also observed in some soybean *QQS* expressers. This phenomenon is not unusual for regulatory proteins to correlate with shallow expression levels (Nagaraj et al., 2011). Therefore, only a minimal concentration of *QQS* saturates the *QQS* receptor (Li et al., 2009), and any increases over this concentration do not affect metabolite content. There might be other possible explanations for a lack of strong linear correlation between levels of *QQS* transcript and metabolite composition, such as *QQS* translational efficiency or stability or the effectiveness of the *QQS* protein to biochemically express its function or post-translational modification is limiting. Indeed, various post-translational regulatory mechanisms can come into play, as have been described for other transgenes (Lillo et al., 2004).

The expression of the *QQS* gene into cassava plants increased leaf protein until 1.36% in line R (LA) L2 and root protein until 17.02% for the same line. Moreover, leaf-soluble total carbohydrate increased to 51.76% in line R’’’(G) L2, and root-soluble total carbohydrate increased to 46.75% in line R(F). For line R7 (F), a low expression in leaves caused a decrease in leaf protein content by 9% while root protein, leaf, and root carbohydrate increased respectively by 13.03%, 34.29%, and 46.75%. A strong correlation (0.9) was found between root protein and total root carbohydrate accumulation. No correlation was found between the level of expression and chemical compound accumulation. Morphological observation has revealed a positive impact of *QQS* expression in cassava, giving a more vigorous plant with larger leaves, a stick stem, and enhanced biomass production. Our findings will be interesting for farmers and agriculture if the biochemical profiling results are confirmed and recapitulated in tuberous roots, stems, and leaves of mature transgenic cassava when grown in the field. Therefore, the novel cassava developed in this project will contribute to ensuring a well-balanced protein diet for the 800 million people in Asia, Africa, and Latin America who rely on the starch crop cassava as their staple food. The capability of *QQS* to increase the soluble total carbohydrate content and, consequently, starch accumulation, if validated in these transgenic mature plants, can be explored by starch and bioethanol producers. *QQS* has also been proven to decrease plant oil accumulation. To validate the molecular mechanism of *QQS* in transgenic cassava plants, the two-yeast hybrid system could be used in further experimentation to see if *QQS* will bind to the conserved transcription factor, nuclear factor Y, subunit C4 (NF-YC4), which is a ubiquitous factor modulating plant development. In terms of safety regarding genetically modified food (GMO), to this day, no concrete identification of toxicity was found in GMOs. Nevertheless, investigations should be done on transgenic *QQS* plants to ensure the safety of crops. It will be interesting to study how *QQS* impacts carbon reparation in plants and, therefore, causes variations in plant chemical composition and vigor. Western blot can be done for protein detection, and Southern blot can be used to check the integration of *QQS* into the cassava genome and evaluate transcript copy number. In addition, the model cassava cultivar (cv 60444), which is not a preferred cultivar for farmers in Africa (Chavarriaga-Aguirre et al., 2016) was used in this study; if the results observed are confirmed on tissues from matured plants, the new traits should be transferred to the consumers and farmers preferred cultivar such as local African cassava landrace variety TME 204 used for nutritional trait enhancement through biotechnology (Narayanan et al., 2019). Finally, a more robust biotechnological approach could be designed to simultaneously combine the expression of *the QQS and AmA1 (Amaranth Albumin)* gene to drive a significant increase in the total protein content in transgenic cassava tubers. Studies demonstrated that tuber-specific expression of *AmA1* (Amaranth Albumin 1), a seed storage protein in transgenic potato tubers, revealed up to 60% increase in total protein content (Chakraborty et al., 2010). Furthermore, a comparative protein profiling of this transgenic potato stably expressing the AmA1 gene unveils proteome rebalancing as a molecular mechanism driving increased protein content in these transgenic potato tubers (Chakraborty et al., 2010). *In vitro* and *In vivo* studies on experimental animals should also be attempted to demonstrate that *QQS* transgenic proteins in transgenic cassava tubers are safe for human consumption.

## Data Availability

The original contributions presented in the study are publicly available. This data can be found here: NCBI, PQ790632, PQ790633 and PQ790634.
