# Supplementary material for: Constitutive overexpression of Qui-Quine Starch gene simultaneously improves starch and protein content in bioengineered cassava (Manihot esculenta Crantz)
Source: Front Plant Sci. 2025 Feb 24;15:1442324. doi: 10.3389/fpls.2024.1442324 (PMC11891011; doi:10.3389/fpls.2024.1442324)
Supplement: Supplementary file 1 [file Presentation1.pptx]

## Slide 1
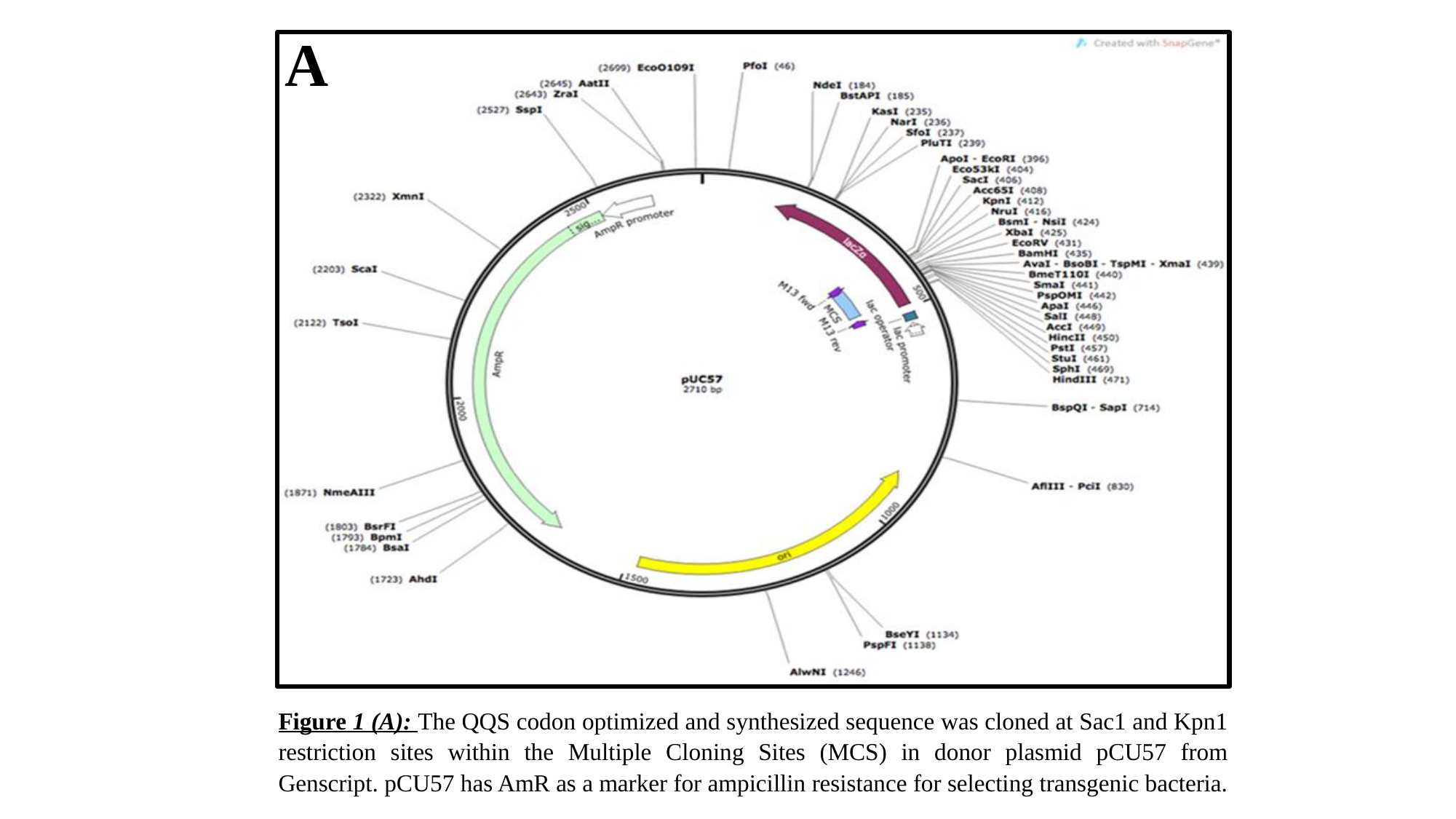

A
Figure 1 (A): The QQS codon optimized and synthesized sequence was cloned at Sac1 and Kpn1 restriction sites within the Multiple Cloning Sites (MCS) in donor plasmid pCU57 from Genscript. pCU57 has AmR as a marker for ampicillin resistance for selecting transgenic bacteria.

## Slide 2
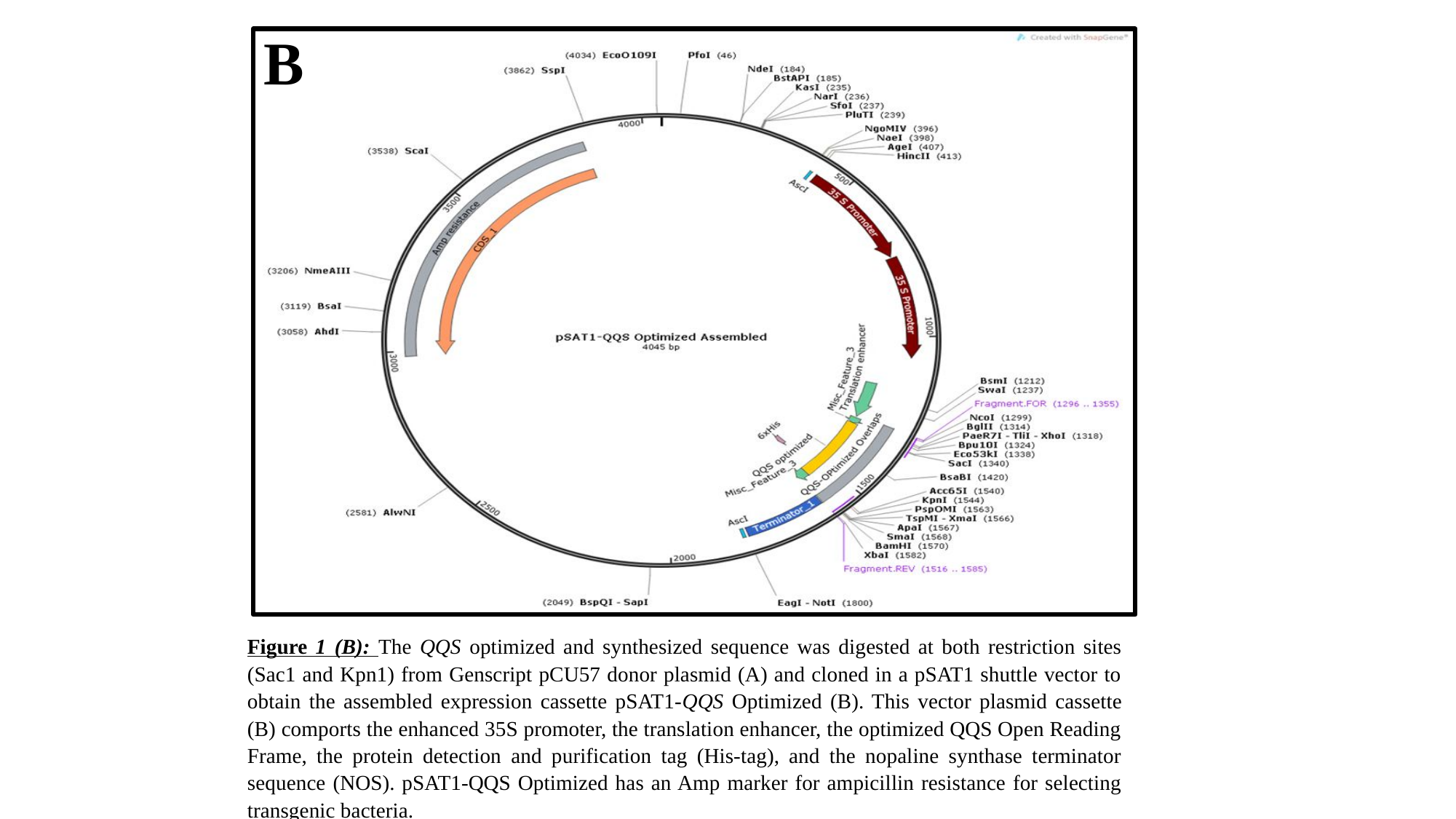

B
Figure 1 (B): The QQS optimized and synthesized sequence was digested at both restriction sites (Sac1 and Kpn1) from Genscript pCU57 donor plasmid (A) and cloned in a pSAT1 shuttle vector to obtain the assembled expression cassette pSAT1-QQS Optimized (B). This vector plasmid cassette (B) comports the enhanced 35S promoter, the translation enhancer, the optimized QQS Open Reading Frame, the protein detection and purification tag (His-tag), and the nopaline synthase terminator sequence (NOS). pSAT1-QQS Optimized has an Amp marker for ampicillin resistance for selecting transgenic bacteria.

## Slide 3
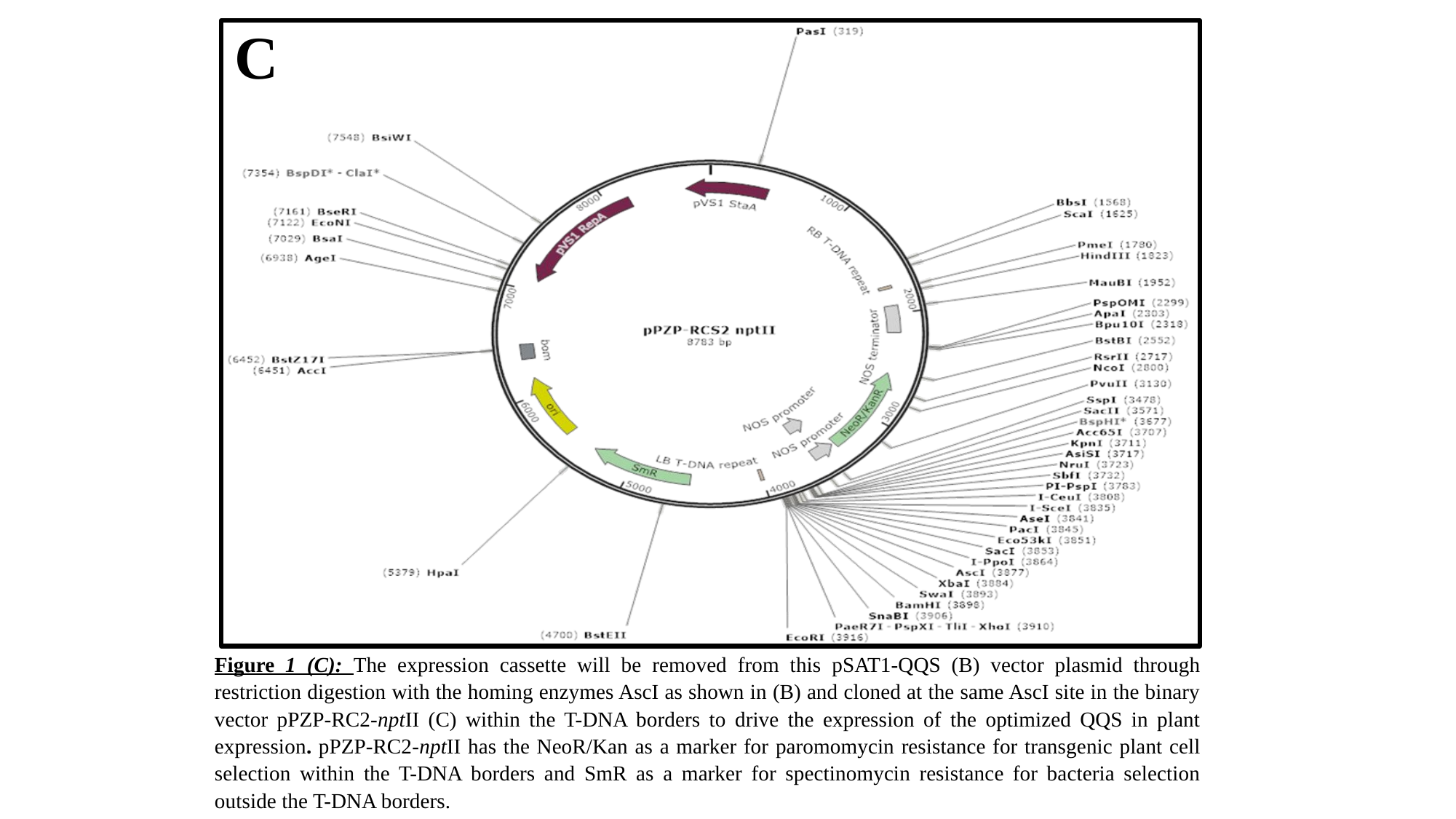

C
Figure 1 (C): The expression cassette will be removed from this pSAT1-QQS (B) vector plasmid through restriction digestion with the homing enzymes AscI as shown in (B) and cloned at the same AscI site in the binary vector pPZP-RC2-nptII (C) within the T-DNA borders to drive the expression of the optimized QQS in plant expression. pPZP-RC2-nptII has the NeoR/Kan as a marker for paromomycin resistance for transgenic plant cell selection within the T-DNA borders and SmR as a marker for spectinomycin resistance for bacteria selection outside the T-DNA borders.

## Slide 4
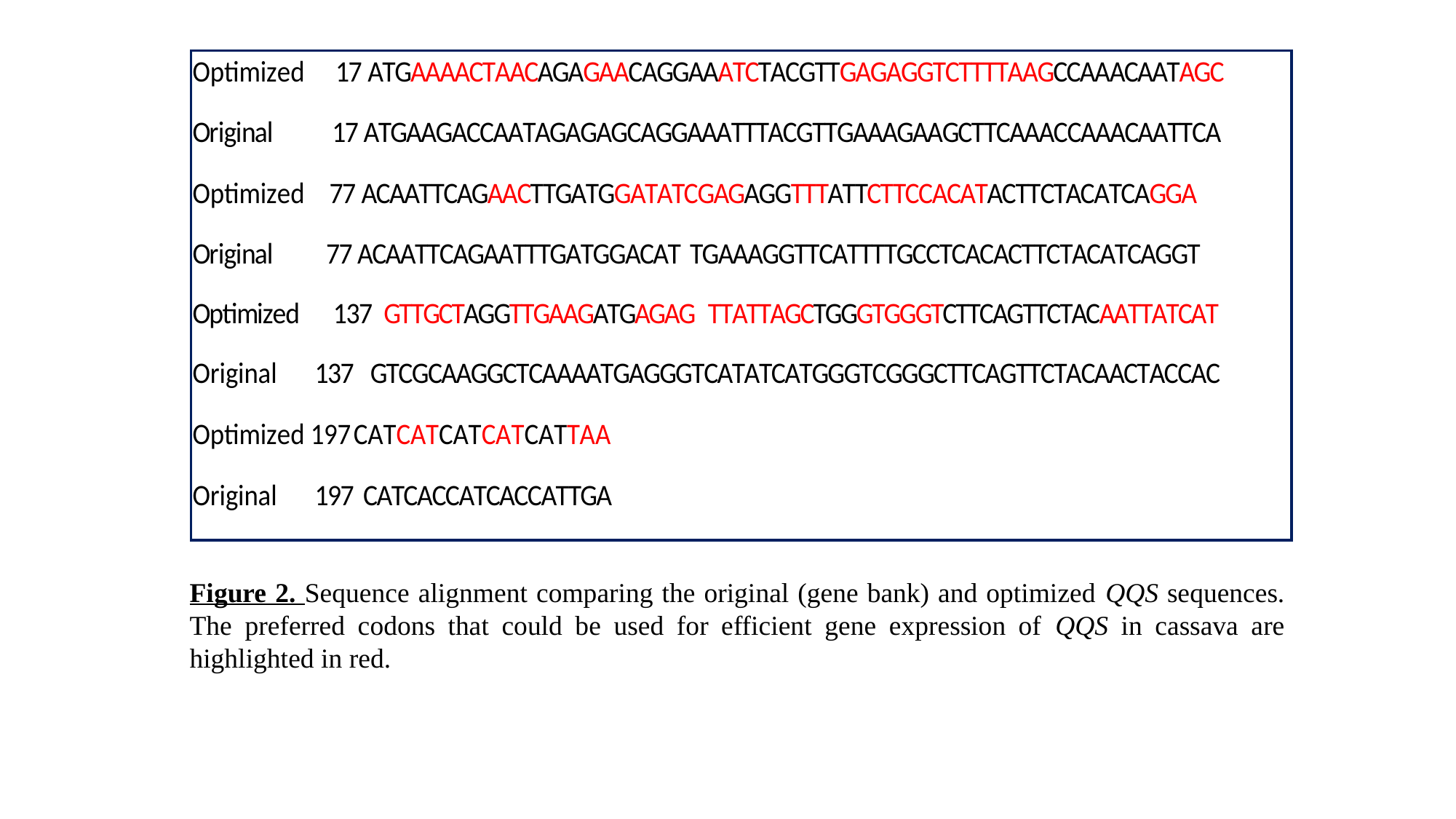

Figure 2. Sequence alignment comparing the original (gene bank) and optimized QQS sequences. The preferred codons that could be used for efficient gene expression of QQS in cassava are highlighted in red.
