# Supplementary material for: Constitutive overexpression of Qui-Quine Starch gene simultaneously improves starch and protein content in bioengineered cassava (Manihot esculenta Crantz)
Source: Front Plant Sci. 2025 Feb 24;15:1442324. doi: 10.3389/fpls.2024.1442324 (PMC11891011; doi:10.3389/fpls.2024.1442324)
Supplement: Supplementary file 2 [file Table1.docx]

**Table 1**. List of gene-specific primers designed for molecular cloning of *QQS* into pSAT1 modular vector and verification of insertion of *QQS* expression cassette prior to subsequent assembly into pPZP-RC2-*npt*II binary vector for plant expression.

| Gene or gene fragment | Sense primer | Antisense primer |
| --- | --- | --- |
| Optimized *QQS* | *aaggaaaggagagctcatga* | *tcctttccttggtaccttaa* |
| Gibson/Gblock of Optimized *QQS* | *tagccatggtccggactcagatctcgagc* | *tagactaggtggatcccgggcccgcggtactc* |
| *QQS* expression cassette fragment check in pSAT1 | *taaggaaaggagagctcatgaaaactaa*  *cag* | *ctttccttggtaccttaatgatgatgatgatgatg*  *ata* |
| *NPTII* | *Atggggattgaacaagatggattgc* | *gaagaactcgtcaagaaggcgatag* |

**Table 2.** Types and composition of media used for cassava regeneration and genetic transformation. MS (MS with vitamins; Murashige & Skoog, 1962), GD (Greshoff & Doy, 1972) composition is based on phytotechnology laboratories (https://phytotechlab.com/), BAP (6-Benzylaminopurine), NAA (Naphthalene acetic acid). Media were solidified by phytagel (0.22%) for CBM and Noble Agar (0.8%) for all other media. pH (5.8).

| Media | Composition | purpose | Reference |
| --- | --- | --- | --- |
| CBM | 4.43g/L MS, 2% (w/v) sucrose, 2 mM  CuSO_4_ | Rooting and *in vitro*  propagation of plantlets | Taylor *et al*. 2001 ; Hankoua *et al*., 2006 Taylor *et al*., 2012. |
| MS2-P50 | 4.43g/L MS, 2% (w/v) sucrose, 50 µM picloram, 2 mM CuSO_4_ | Initiation and maintenance of primary, secondary, and cyclic somatic embryos | Taylor *et al*. 2001 ; Hankoua *et al*., 2006 ; Taylor *et al*., 2012. |
| GD2-50P | GD, 2% (w/v) sucrose, 2 mM CuSO_4,_  50 µM picloram | initiation of FEC | Taylor *et al*. 2001 ; Hankoua *et al*., 2006 ; Taylor *et al*., 2012. |
| GD6-50P | GD, 6% (w/v) sucrose, 2 mM CuSO_4,_  50 µM picloram | Maintenance of homogenous and highly proliferating FEC | Taylor *et al*. 2001 ; Hankoua *et al*., 2006 ; Taylor *et al*., 2012. |
| EMM | 4.43g/L MS, 2% (w/v) sucrose, 0.5 mg/L +45 µM paromomycin + 04 mg/L NAA | regeneration of  transgenic somatic embryos | Taylor *et al*. 2001 ; Hankoua *et al*., 2006 ; Taylor *et al*., 2012. |
| EGM | 4.43g/L MS, 2% (w/v) sucrose,  0.4mg/L BAP | Shoot induction from emerged somatic embryos. | Taylor *et al*. 2001 ; Hankoua *et al*., 2006 ; Taylor *et al*., 2012. |

**Table 3**. A list of primers was used for quantitative (qRT-PCR) to study the codon-optimized *QQS* gene expression from non-*QQS* expresser control plants and transgenic cassava tissues that expressed the *QQS* gene. The Cassava α-tubulin gene (TC3055) was used as an internal control.

| Gene | Sense primer | Antisense primer |
| --- | --- | --- |
| *codon-optimized QQS* | *gaacaggaaatctacgttga* | *ccacccagctaataactctc* |
| *Manihot esculenta*  *α-tubulin (TC3055)* | caagtgcgatcctcgacatg | gataccgcacttgaacccag |

**Table 4***:* Summary of results of amino-acid composition for leaf, root, and stem biomass of each stable transgenic line and wild-type non-transgenic controls. For each amino acid, the mean value of each sample was plotted to obtain Figure 10.

| **Sample type** | BF-1 | | BF-2 | | BF-3 | | BF-4 | | BF-5 | | BF-6 | | BF-7 | |
| --- | --- | --- | --- | --- | --- | --- | --- | --- | --- | --- | --- | --- | --- | --- |
| **AA** | mean | s.d. | mean | s.d. | mean | s.d. | mean | s.d. | mean | s.d. | mean | s.d. | Mean | s.d. |
| ALA | 6.85 | 0.29 | 2.28 | 0.05 | 2.77 | 0.13 | 7.61 | 0.23 | 7.34 | 0.49 | 2.03 | 0.05 | 3.00 | 0.73 |
| ARG | 8.18 | 0.44 | 3.04 | 0.04 | 2.47 | 0.24 | 9.00 | 0.35 | 8.57 | 0.57 | 2.46 | 0.04 | 2.91 | 0.90 |
| AASX | 11.75 | 0.67 | 4.24 | 0.09 | 4.92 | 0.31 | 13.41 | 0.52 | 12.63 | 0.90 | 3.94 | 0.06 | 4.61 | 0.94 |
| GLX | 14.79 | 0.87 | 8.09 | 0.35 | 6.74 | 0.48 | 16.94 | 0.64 | 15.36 | 1.16 | 5.67 | 0.08 | 6.54 | 1.44 |
| GLY | 5.95 | 0.47 | 1.62 | 0.03 | 2.10 | 0.11 | 6.59 | 0.21 | 6.37 | 0.55 | 1.64 | 0.04 | 2.19 | 0.50 |
| HIS | 2.92 | 0.17 | 0.93 | 0.03 | 1.12 | 0.05 | 3.23 | 0.06 | 3.09 | 0.18 | 0.84 | 0.03 | 1.12 | 0.17 |
| ILE | 5.88 | 0.29 | 1.86 | 0.06 | 2.49 | 0.11 | 6.57 | 0.13 | 6.34 | 0.36 | 1.89 | 0.07 | 2.43 | 0.34 |
| LEU | 10.91 | 0.55 | 2.88 | 0.11 | 3.92 | 0.18 | 12.09 | 0.26 | 11.54 | 0.64 | 2.93 | 0.11 | 3.93 | 0.55 |
| LYS | 5.67 | 0.47 | 2.41 | 0.09 | 2.85 | 0.16 | 7.34 | 0.05 | 6.43 | 0.30 | 2.65 | 0.07 | 2.91 | 0.43 |
| MET | 2.20 | 0.08 | 0.49 | 0.04 | 0.80 | 0.09 | 2.31 | 0.04 | 1.91 | 0.57 | 0.29 | 0.04 | 0.46 | 0.40 |
| PHE | 7.11 | 0.32 | 1.80 | 0.09 | 2.66 | 0.11 | 7.84 | 0.14 | 7.55 | 0.42 | 1.69 | 0.08 | 2.55 | 0.36 |
| PRO | 7.04 | 0.22 | 1.67 | 0.05 | 2.17 | 0.10 | 7.67 | 0.42 | 6.66 | 0.92 | 1.57 | 0.06 | 2.46 | 0.26 |
| SER | 5.40 | 0.32 | 1.98 | 0.04 | 2.63 | 0.18 | 6.12 | 0.25 | 5.76 | 0.44 | 1.80 | 0.06 | 2.59 | 0.52 |
| THR | 5.74 | 0.29 | 1.79 | 0.05 | 2.13 | 0.12 | 6.36 | 0.21 | 6.11 | 0.40 | 1.80 | 0.03 | 2.18 | 0.49 |
| TYR | 5.16 | 0.24 | 1.33 | 0.08 | 1.91 | 0.09 | 5.68 | 0.17 | 5.39 | 0.31 | 1.19 | 0.08 | 1.81 | 0.23 |
| VAL | 6.64 | 0.32 | 2.17 | 0.04 | 2.79 | 0.11 | 7.51 | 0.16 | 7.20 | 0.44 | 2.02 | 0.06 | 2.74 | 0.45 |

B

| **Sample type** | BF-8 | | BF-9 | | BF-10 | | BF-11 | | BF-12 | | BF-13 | | BF-14 | |
| --- | --- | --- | --- | --- | --- | --- | --- | --- | --- | --- | --- | --- | --- | --- |
| **AA** | mean | s.d. | mean | s.d. | mean | s.d. | mean | s.d. | mean | s.d. | mean | s.d. | mean | s.d. |
| ALA | 7.72 | 0.04 | 3.27 | 0.88 | 2.81 | 0.40 | 6.88 | 0.73 | 2.09 | 0.52 | 2.90 | 0.13 | 5.53 | 1.25 |
| ARG | 9.10 | 0.28 | 3.39 | 1.06 | 2.46 | 0.17 | 8.22 | 0.83 | 2.77 | 1.11 | 2.77 | 0.04 | 6.14 | 1.22 |
| ASX | 13.51 | 0.10 | 5.43 | 1.33 | 4.40 | 0.32 | 12.06 | 1.32 | 4.09 | 1.33 | 4.97 | 0.22 | 10.06 | 2.28 |
| GLX | 16.80 | 0.20 | 8.36 | 2.18 | 5.85 | 0.57 | 14.87 | 1.57 | 7.97 | 3.88 | 6.92 | 0.39 | 12.76 | 2.61 |
| GLY | 6.80 | 0.04 | 2.06 | 0.43 | 1.93 | 0.11 | 5.91 | 0.63 | 1.57 | 0.43 | 2.09 | 0.08 | 4.77 | 1.05 |
| HIS | 3.33 | 0.08 | 1.09 | 0.24 | 1.01 | 0.08 | 2.90 | 0.30 | 0.92 | 0.26 | 1.21 | 0.06 | 2.27 | 0.50 |
| ILE | 6.78 | 0.12 | 2.33 | 0.51 | 2.22 | 0.16 | 5.83 | 0.63 | 1.79 | 0.41 | 2.53 | 0.10 | 4.77 | 1.03 |
| LEU | 12.39 | 0.24 | 3.67 | 0.73 | 3.52 | 0.26 | 10.91 | 1.17 | 2.85 | 0.68 | 3.95 | 0.16 | 8.65 | 1.82 |
| LYS | 7.00 | 0.28 | 3.24 | 0.86 | 2.60 | 0.21 | 5.47 | 0.54 | 2.23 | 0.60 | 2.98 | 0.12 | 4.68 | 1.01 |
| MET | 2.32 | 0.06 | 0.42 | 0.04 | 0.59 | 0.05 | 2.46 | 0.25 | 0.47 | 0.11 | 0.65 | 0.02 | 1.64 | 0.15 |
| PHE | 8.11 | 0.21 | 2.21 | 0.38 | 2.31 | 0.14 | 7.17 | 0.77 | 1.80 | 0.38 | 2.59 | 0.09 | 5.65 | 1.16 |
| PRO | 8.31 | 0.23 | 1.94 | 0.40 | 2.37 | 0.59 | 7.00 | 0.85 | 1.50 | 0.31 | 2.21 | 0.10 | 5.56 | 1.49 |
| SER | 6.15 | 0.02 | 2.37 | 0.53 | 2.30 | 0.21 | 5.27 | 0.56 | 1.86 | 0.45 | 2.60 | 0.11 | 4.46 | 0.89 |
| THR | 6.43 | 0.04 | 2.26 | 0.52 | 1.98 | 0.19 | 5.75 | 0.63 | 1.70 | 0.47 | 2.22 | 0.07 | 4.59 | 0.93 |
| TYR | 5.86 | 0.11 | 1.52 | 0.35 | 1.66 | 0.40 | 5.20 | 0.56 | 1.45 | 0.31 | 1.97 | 0.07 | 3.87 | 0.74 |
| VAL | 7.73 | 0.08 | 2.59 | 0.52 | 2.48 | 0.15 | 6.64 | 0.71 | 2.08 | 0.52 | 2.84 | 0.12 | 5.42 | 1.22 |

C

| **Sample type** | BF-15 | | BF-16 | | BF-17 | | BF-18 | | BF-19 | | BF-20 | | BF-21 | | BF-22 | |
| --- | --- | --- | --- | --- | --- | --- | --- | --- | --- | --- | --- | --- | --- | --- | --- | --- |
| **AA** | mean | s.d. | mean | s.d. | mean | s.d. | Mean | s.d. | mean | s.d. | mean | s.d. | mean | s.d. | mean | s.d. |
| ALA | 2.17 | 0.12 | 2.38 | 0.55 | 6.09 | 1.87 | 1.68 | 0.17 | 2.28 | 0.19 | 6.80 | 0.51 | 2.42 | 0.09 | 2.53 | 0.19 |
| ARG | 2.63 | 0.11 | 2.30 | 0.54 | 7.17 | 2.35 | 1.89 | 0.20 | 2.63 | 0.36 | 7.46 | 1.12 | 2.97 | 0.22 | 2.60 | 0.21 |
| ASX | 4.30 | 0.39 | 3.97 | 0.95 | 10.91 | 3.30 | 3.25 | 0.33 | 3.90 | 0.34 | 12.29 | 0.87 | 4.78 | 0.28 | 4.34 | 0.45 |
| GLX | 7.88 | 1.04 | 5.29 | 1.19 | 13.92 | 3.67 | 4.89 | 0.47 | 5.14 | 0.37 | 15.73 | 0.90 | 8.70 | 0.46 | 5.77 | 0.83 |
| GLY | 1.58 | 0.09 | 1.74 | 0.44 | 5.31 | 1.61 | 1.27 | 0.13 | 1.70 | 0.15 | 5.95 | 0.45 | 1.79 | 0.08 | 1.84 | 0.16 |
| HIS | 0.88 | 0.04 | 0.93 | 0.24 | 2.63 | 0.82 | 0.70 | 0.07 | 0.96 | 0.10 | 2.94 | 0.18 | 0.97 | 0.04 | 1.04 | 0.09 |
| ILE | 1.78 | 0.08 | 2.04 | 0.52 | 5.31 | 1.56 | 1.47 | 0.15 | 2.07 | 0.21 | 5.84 | 0.50 | 2.00 | 0.07 | 2.20 | 0.18 |
| LEU | 2.75 | 0.11 | 3.22 | 0.82 | 9.74 | 2.95 | 2.31 | 0.25 | 3.30 | 0.31 | 10.60 | 0.94 | 3.11 | 0.09 | 3.44 | 0.29 |
| LYS | 2.32 | 0.12 | 2.35 | 0.61 | 5.24 | 1.86 | 1.67 | 0.17 | 2.20 | 0.25 | 5.62 | 0.63 | 2.63 | 0.08 | 2.66 | 0.24 |
| MET | 0.44 | 0.03 | 0.49 | 0.10 | 1.91 | 0.35 | 0.42 | 0.06 | 0.29 | 0.27 | 1.97 | 0.22 | 0.50 | 0.02 | 0.46 | 0.23 |
| PHE | 1.71 | 0.06 | 2.15 | 0.57 | 6.36 | 1.96 | 1.44 | 0.16 | 2.10 | 0.22 | 6.90 | 0.64 | 1.90 | 0.07 | 2.26 | 0.19 |
| PRO | 1.60 | 0.26 | 2.11 | 0.80 | 5.95 | 2.15 | 1.22 | 0.13 | 2.24 | 0.17 | 6.89 | 0.49 | 1.65 | 0.08 | 1.93 | 0.17 |
| SER | 1.89 | 0.11 | 2.17 | 0.53 | 5.00 | 1.45 | 1.42 | 0.15 | 2.15 | 0.20 | 5.52 | 0.42 | 2.09 | 0.11 | 2.29 | 0.22 |
| THR | 1.75 | 0.11 | 1.82 | 0.44 | 5.09 | 1.50 | 1.32 | 0.12 | 1.80 | 0.16 | 5.64 | 0.43 | 1.88 | 0.07 | 1.94 | 0.15 |
| TYR | 1.38 | 0.19 | 1.56 | 0.52 | 4.50 | 1.46 | 1.16 | 0.13 | 1.70 | 0.18 | 4.76 | 0.65 | 1.67 | 0.11 | 1.68 | 0.15 |
| VAL | 2.09 | 0.14 | 2.31 | 0.59 | 5.98 | 1.77 | 1.66 | 0.18 | 2.28 | 0.22 | 6.72 | 0.54 | 2.36 | 0.08 | 2.47 | 0.20 |

**Table 5**: Detailed information on the genes and biological processes

| **Display name** | **UniProt ID** | **Description** | **Interactor score** |
| --- | --- | --- | --- |
| QQS | Q3E7K4 | Protein QQS; Involved in regulating carbon and nitrogen allocation to starch and protein. | query |
| NFYC4 | Q9FMV5 | Nuclear transcription factor Y subunit C-4; Stimulates the transcription of various genes by recognizing and binding to a CCAAT motif in promoters (By similarity). Involved in the abscisic acid (ABA) signaling pathway. | 0.983 |
| B3H5U3_ARATH | B3H5U3 | Transmembrane protein | 0.854 |
| IDD14 | Q9C9X7 | Protein indeterminate-domain 14; Transcription factor regulating starch metabolism by binding directly to the promoter of QQS . The IDD14beta isoform attenuates the transcription factor activity by competitively forming heterodimers with reduced DNA-binding capacity. Regulates lateral organ morphogenesis and gravitropic responses . Has a redundant role with IDD16 in directing leaf and floral organ morphogenesis . Involved in the establishment of auxin gradients through the regulation of auxin biosynthesis and transport . | 0.805 |
| F15H21.8 | Q9C7W0 | At1g64360 | 0.796 |
| AtEWR1 | A0A1I9LNG9 | Transmembrane protein | 0.796 |
| Q93ZI5_ARATH | Q93ZI5 | At1g58243/At1g58243 | 0.784 |
| MWF20.20 | Q9LSW4 | Uncharacterized protein | 0.784 |
| Q0V817_ARATH | Q0V817 | At4g12005 | 0.784 |
| F4IZ43_ARATH | F4IZ43 | Uncharacterized protein | 0.784 |
| B3H5B2_ARATH | B3H5B2 | Uncharacterized protein | 0.784 |
| Q1G3A0_ARATH | Q1G3A0 | Uncharacterized protein | 0.784 |
| T24M8.5 | O81514 | Putative glucose-6-phosphate/phosphate-translocator-like protein 1 | 0.76 |
| B3H5F1_ARATH | B3H5F1 | Uncharacterized protein | 0.757 |
| CYP21-2 | Q8L8W5 | Peptidyl-prolyl cis-trans isomerase CYP21-2; PPIases accelerate the folding of proteins. It catalyses the cis-trans isomerization of proline imidic peptide bonds in oligopeptides (By similarity). | 0.757 |
| T19G15.120 | F4JZS0 | Transmembrane protein | 0.741 |
| F4JT34_ARATH | F4JT34 | Uncharacterized protein | 0.739 |
| F4J7U9_ARATH | F4J7U9 | Eukaryotic aspartyl protease family protein | 0.739 |
| F8M21.250 | Q9LXE7 | Transmembrane protein | 0.739 |
| Q56YJ7_ARATH | Q56YJ7 | Transmembrane protein | 0.739 |
| B3H4S9_ARATH | B3H4S9 | Uncharacterized protein | 0.739 |
| F13M14.29 | Q9CAE7 | Putative F-box protein At3g10430 | 0.739 |
| FOLT1 | Q7XA87 | Folate transporter 1, chloroplastic; Mediates folate import into chloroplast. ; Belongs to the mitochondrial carrier (TC 2.A.29) family. | 0.72 |
| Q94BZ4_ARATH | Q94BZ4 | At2g34655 | 0.719 |
| F7J8.60 | F4K7X9 | Beta-galactosidase related protein | 0.719 |
| POLAR | Q6NQ99 | Protein POLAR LOCALIZATION DURING ASYMMETRIC DIVISION AND REDISTRIBUTION; Regulates asymmetric cell division (ACD), especially in stomatal-lineage cells. Acts as a stomatal lineage scaffold which regulates subcellular localization and transient polarization of kinases (e.g. ASK7/BIN2 and ASK3/SK12) involved in ACD in a BASL-dependent manner. Promotes the differentiation of both pavement cells and stomata. | 0.719 |
| SS3 | F4IAG2 | Starch synthase 3, chloroplastic/amyloplastic; Involved in synthesizing glycan chains within amylopectin in leaves. May play a regulatory role in the control of starch accumulation in plastids; Belongs to the glycosyltransferase 1 family. Bacterial/plant glycogen synthase subfamily. | 0.719 |
| PI4KG3 | Q9FNF8 | Phosphatidylinositol 4-kinase gamma 3; The phosphorylation of phosphatidylinositol (PI) to PI4P is the first committed step in the generation of phosphatidylinositol 4,5- bisphosphate (PIP2), a precursor of the second messenger inositol 1,4,5-trisphosphate (InsP3); Belongs to the PI3/PI4-kinase family. Type II PI4K subfamily. | 0.717 |
| M1180_ARATH | P92548 | Uncharacterized mitochondrial protein AtMg01180 | 0.696 |
| F4J7V3_ARATH | F4J7V3 | Retrotransposon ORF-1 protein | 0.696 |
| F26G5_20 | Q9LXQ5 | Glycosyl hydrolase family 35 protein | 0.695 |
| Q9LTY3_ARATH | Q9LTY3 | Cysteine/Histidine-rich C1 domain family protein | 0.695 |
| F4IMB6_ARATH | F4IMB6 | Uncharacterized protein | 0.695 |
| EXL1-2 | Q9C6E4 | Protein EXORDIUM-like 1; May play a role in a brassinosteroid-dependent regulatory pathway that controls growth and development under low carbon and energy availability; Belongs to the EXORDIUM family. | 0.653 |
| IDD16 | Q9FRH4 | Protein indeterminate-domain 16; Transcription factor regulating lateral organ morphogenesis and gravitropic responses. Has a redundant role with IDD14 in directing leaf and floral organ morphogenesis. Acts cooperatively with IDD15 to control silique and branche orientation. Involved in the establishment of auxin gradients through the regulation of auxin biosynthesis and transport. | 0.6 |
| F19B15.50 | Q9SZD2 | Putative glycine-rich protein 5 | 0.599 |
| GDU6 | Q3EAV6 | Protein GLUTAMINE DUMPER 6; Probable subunit of an amino acid transporter involved in regulating the amino acid metabolism. Stimulates amino acid export by activating nonselective amino acid facilitators. | 0.598 |
| F10A16.1 | Q9M9M3 | Defensin-like protein 205 | 0.598 |
| PIRL2 | Q9LRV8 | Plant intracellular Ras-group-related LRR protein 2; Leucine-rich repeat protein that likely mediates protein interactions, possibly in signal transduction. ; Belongs to the SHOC2 family. | 0.597 |
| K2N11.2 | Q9FH44 | Ulp1 protease family protein | 0.597 |
| HDG6 | Q9FVI6 | Homeobox-leucine zipper protein HDG6; Probable transcription factor involved in regulating flowering time through the photoperiod flowering pathway. May repress FT. | 0.59 |
| SIS8 | Q9C9U5 | Probable serine/threonine-protein kinase SIS8 Acts as a negative regulator of salt tolerance. Mediates sugar response during early seedling development; Belongs to the protein kinase superfamily. Ser/Thr protein kinase family. | 0.575 |
| SDC | Q3EBY8 | F-box protein At2g17690; Involved in heat stress response. Contributes to recovery from heat stress. | 0.549 |
| PURU2 | F4JP46 | Formyltetrahydrofolate deformylase 2, mitochondrial; Deformylase involved in photorespiration. Prevents excessive accumulation of 5-formyl tetrahydrofolate (THF), a potent inhibitor of the Gly decarboxylase/Ser hydroxymethyltransferase complex. | 0.548 |
| NAD-ME1-2 | Q9T0H6 | Late embryogenesis abundant protein (LEA) family protein | 0.548 |
| CMT3 | Q94F88 | DNA (cytosine-5)-methyltransferase CMT3; Involved in the CpXpG methylation and in gene silencing. Methylates preferentially transposon-related sequences. Functionally redundant to DRM1/DRM2 to maintain non-CpG methylation. Involved in RNA-directed DNA methylation. ECO:0000269\|PubMed:11459824, ECO:0000269\|PubMed:11790305,; Belongs to the class I-like SAM-binding methyltransferase superfamily. C5-methyltransferase family. | 0.543 |
| UGT74E1 | P0C7P7 | UDP-glycosyltransferase 74E1; Belongs to the UDP-glycosyltransferase family. | 0.54 |
| CRK31 | Q9LDM5 | Putative cysteine-rich receptor-like protein kinase 31 | 0.54 |
| Q3EBD7_ARATH | Q3EBD7 | Expressed protein | 0.514 |
| ROH1 | Q9CAK4 | Protein ROH1; Required for seed coat mucilage deposition. | 0.513 |
| MAIL1 | Q9SK32 | Protein MAIN-LIKE 1; Acts as an important factor for cell fate determination and maintenance throughout plant development. Required for the organization of the root apical meristem (RAM) and the shoot apical meristem (SAM). Required to maintain genome stability and cell division activity in meristematic cells. | 0.482 |
| SGR5 | F4IPE3 | Zinc finger protein SHOOT GRAVITROPISM 5; Transcription factor involved in inflorescence stems gravitropism, probably by regulating starch accumulation in amyloplasts of graviperceptive cells. Required for stem circumnutation movements. Regulates lateral organ morphogenesis and gravitropic responses . Acts cooperatively with IDD16 to control silique and branche orientation . Involved in the establishment of auxin gradients through the regulation of auxin biosynthesis and transport . ECO:0000269\|PubMed:18259878, ECO:0000269\|PubMed:24039602, | 0.478 |
| LURE1.1 | Q4VP09 | Protein LURE 1.1; Pollen tube attractants guiding pollen tubes to the ovular micropyle; Belongs to the DEFL family. | 0.436 |
| CRK41 | O23081 | Cysteine-rich receptor-like protein kinase 41 | 0.431 |
| F14N22.17 | Q9SIN3 | Late embryogenesis abundant domain-containing protein / LEA domain-containing protein | 0.431 |
| TED7 | Q9FI79 | Protein TRACHEARY ELEMENT DIFFERENTIATION-RELATED 7; Essential protein. Involved in the secondary cell wall (SCW) formation of vessel elements (e.g. protoxylem and metaxylem), thus promoting tracheary element (TE) differentiation. | 0.431 |
| WRKY46 | Q9SKD9 | Probable WRKY transcription factor 46; Transcription factor involved in regulating osmotic stress responses and stomatal movement. Interacts specifically with the W box (5'-(T)TGAC[CT]-3'), a frequently occurring elicitor-responsive cis-acting element (By similarity). Positive regulator of EDS1-dependent defense against E.amylovora . Together with WRKY70 and WRKY53, promotes resistance to P.syringae, probably by enhancing salicylic acid (SA)- dependent genes. Contributes to the suppression of jasmonic acid (MeJA)-induced expression of PDF1.2 . Together with WRKY54 and WRKY70, promotes brassinosteroid (BR)-regulated plant growth but prevent drought response by modulating gene expression. ECO:0000269\|PubMed:22325892, ECO:0000269\|PubMed:24773321, | 0.431 |
| PAP26 | Q949Y3 | Bifunctional purple acid phosphatase 26; Metallo-phosphoesterase involved in phosphate metabolism. Acid phosphatase activity with phosphoenolpyruvate, inorganic pyrophosphate, phenyl-phosphate and p-nitrophenyl-phosphate as the most effective substrates. No activity with phytic acid, phosphocholine or bis-p-nitrophenyl-phosphate. Has a peroxidase activity at alkaline pH. ; Belongs to the metallophosphoesterase superfamily. Purple acid phosphatase family. | 0.416 |
| VIP2-3 | Q9FPW4 | Probable NOT transcription complex subunit VIP2; Transcriptional regulator required for Agrobacterium-mediated stable genetic transformation by T-DNA integration in host genome, but not for T-DNA transient expression; Belongs to the CNOT2/3/5 family. | 0.404 |
| DIR11 | Q67YM6 | Dirigent protein 11; Dirigent proteins impart stereoselectivity on the phenoxy radical-coupling reaction, yielding optically active lignans from two molecules of coniferyl alcohol in the biosynthesis of lignans, flavonolignans, and alkaloids and thus plays a central role in plant secondary metabolism. | 0.4 |
